# Evolving Paradigm of Prothrombin Time Diagnostics with Its Growing Clinical Relevance towards Cardio-Compromised and COVID-19 Affected Population

**DOI:** 10.3390/s21082636

**Published:** 2021-04-09

**Authors:** Anubhuti Saha, Ashutosh Bajpai, Vinay Krishna, Shantanu Bhattacharya

**Affiliations:** 1 Design Program, Indian Institute of Technology, Kanpur 208016, India; anusaha@iitk.ac.in; 2 Microsystems Fabrication Laboratory, Indian Institute of Technology, Kanpur 208016, India; 3 LPS Institute of Cardiology, GSVM Medical College, Kanpur 208002, India; ashutosh.bajpai.ftp@gmail.com (A.B.); vinaycvts@gmail.com (V.K.)

**Keywords:** prothrombin time (PT), international normalized ratio (INR), hemostasis, thromboembolism, coagulopathy, COVID-19, COVID associated coagulopathy (CAC), point of care (PoC)

## Abstract

Prothrombin time (PT) is a significant coagulation (hemostasis) biomarker used to diagnose several thromboembolic and hemorrhagic complications based on its direct correlation with the physiological blood clotting time. Among the entire set of PT dependents, candidates with cardiovascular ailments are the major set of the population requiring lifelong anticoagulation therapy and supervised PT administration. Additionally, the increasing incidence of COVID affected by complications in coagulation dynamics has been strikingly evident. Prolonged PT along with sepsis-induced coagulopathy (SIC score > 3) has been found to be very common in critical COVID or CAC-affected cases. Considering the growing significance of an efficient point-of-care PT assaying platform to counter the increasing fatalities associated with cardio-compromised and coagulation aberrations propping up from CAC cases, the following review discusses the evolution of lab-based PT to point of care (PoC) PT assays. Recent advances in the field of PoC PT devices utilizing optics, acoustics, and mechanical and electrochemical methods in microsensors to detect blood coagulation are further elaborated. Thus, the following review holistically aims to motivate the future PT assay designers/researchers by detailing the relevance of PT and associated protocols for cardio compromised and COVID affected along with the intricacies of previously engineered PoC PT diagnostics.

## 1. Introduction

**Figure 1:**
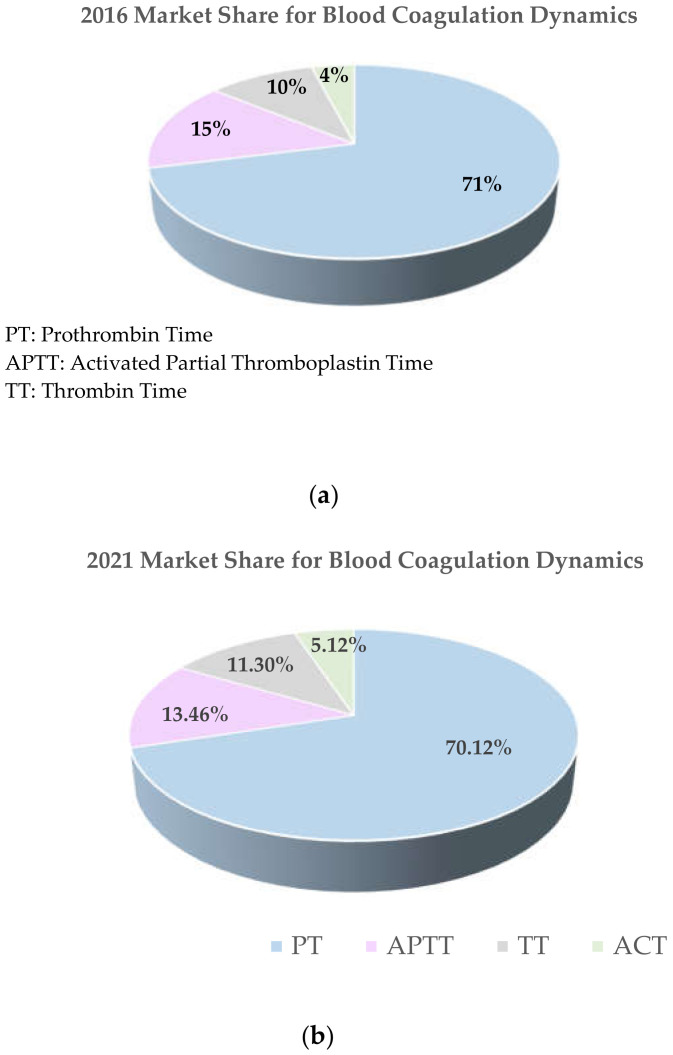
Global coagulation analyzer market share (**a**) in 2016 (**b**) predicted for the year 2021 (Reproduced from the data provided by ref. [26]).

Prothrombin time (PT) is the tissue factor-induced clotting time of plasma blood triggered via an extrinsic pathway to ensure coagulation. PT with associated international normalized ratio (INR) is a standardized coagulation test performed to evaluate several thromboembolic and hemorrhagic disorders. PT test candidates are a part of a highly sensitive set of the population with the following impairments, viz. (i) thromboembolic complications where oral anticoagulation therapy (OAT) is being administered; (ii) critical coagulopathy (such as SIC), hemorrhagic conditions, etc., prompted through hepatic/renal disorders, or diseased states like dengue hemorrhagic fever, etc.; (iii) pre-, peri-, and post-operative conditions (leading sometimes to imbalances in hemostasis); and (iv) COVID-19 associated coagulopathy (CAC) cases with post-infection complications. Among the entire set of PT dependents, patients with cardiovascular disorders (CVD) such as atrial fibrillation, venous thrombosis, pulmonary embolism, stroke, transient ischemic attacks, myocardial infarction, valve prosthesis, etc., are among the main candidates requiring life-long oral therapy to prevent intravascular clot formations. It has been noted that for antithrombotic drugs, the ones which are used for the treatment of cardiovascular ailments formulate 53.1% [1] of the global market share. Thus, the dependency on PT diagnosis platforms of the patients who are cardio-compromised or have coagulopathic aberrations due to diseased states is very high, and this work reviews the state of the art for such diagnostic platform keeping in mind these subjects. Statistically, cardiovascular diseases are the leading cause of death globally, as reported by the World Health Organization (WHO). According to statistical reports by WHO, approximately 31% (17.9 million) of the global deaths are accounted to share the global mortality count due to cardiovascular ailments in 2016 [2]. This global mortality count is expected to grow to more than 23.6 million by 2030 [3]. Annually, an estimated population of 92.1 million US adults bears at least one type of CVD, which is projected to increase up to 43.9% of the US adult population by 2030 as reported by the American Heart Association [4]. Compared to the respective alarming scenario in developed countries, statistically, it has been also reported that globally 80% of CVD deaths are accounted by low- and middle-income countries [3]. This alarming incidence of cardio-compromised population ensures the need for efficient PT diagnosis protocols. In addition to the sector of cardio-compromised population and candidates under OAT, PT tests are also performed to assess hemorrhagic type coagulation aberrations caused due to SIC, Vit K deficiency, renal/hepatic disorders, a person suffering from dengue hemorrhagic fever [5], or the candidates under critical pre-peri-post operative condition [6]. Apart from these predefined diseased conditions, complex coagulopathic conditions exhibited by the growing group of the novel coronavirus affected have been found to be strikingly evident [7]. Early studies made on 183 consecutive patients with novel coronavirus pneumonia (NCP) in Wuhan, China, exhibited significantly abnormal coagulation parameters [8]. High levels of D-dimer and fibrin degradation product (FDP), longer prothrombin time, and activated partial thromboplastin were observed in non-survivors compared to the survivors, among which 71.4% of non-survivors and 0.6% survivors met the criteria of disseminated intravascular coagulation [8]. A striking incidence of thromboembolic events (TEs) with an early manifestation of venous thromboembolism (VTE) has been observed in 30–69% of the COVID-19 intensive care unit (ICU) patients despite standard heparin thromboprophylaxis [9,10]. Studies conducted on 184 ICU patients by Klok et al. also confirmed the very high cumulative incidence of thrombotic complications such as deep vein thrombosis, pulmonary embolism, stroke, and venous thromboembolism in critically ill patients with COVID-19 pneumonia [11]. A significant predisposition of both venous and arterial thromboembolic complications in the COVID affected is considered to be caused due to excessive systemic inflammation and endothelial dysfunction [12,13]. According to real-time WHO statistics, the cumulative number of confirmed COVID-19 cases worldwide as of November 2020 is reported to be over 49.7 million with over 1.2 million deaths globally (since the start of the pandemic) [14,15]. Thus, the respective mortality counts along with the increasing prevalence of COVID-associated coagulopathic complications discussed previously foretells a futuristic increase in incidents of COVID-associated coagulopathy cases. Although the pathophysiology of CAC and the underlying mechanism of its clinical manifestation remains unclear, it seems to provide clinical resemblance with systemic coagulopathies such as sepsis-induced coagulopathy (SIC) or disseminated intravascular coagulation (DIC) to a great extent [12,16]. Venous thromboembolism, deep vein thromboembolism (as observed in 58% of COVID-affected patients), and microangiopathy along with circulatory embolism leading to fatalities such as pulmonary embolism, acute respiratory distress syndrome (ARDS), multi-organ dysfunction syndrome (MODS), etc., are found to be common in CAC cases [7,16,17]. Along with the respective scenario of CAC, the coagulation biomarkers such as PT and D-dimers were found to be a significant indicator of the level of criticality in the COVID-affected patients [18]. Prolonged PT has been found to be directly associated with critical CAC in addition to SIC and MODS [16,19]. Along with PT, prothrombin-associated markers such as prothrombin time activity % (PT-act%) and prothrombin fragment (PF) 1.2 marker were found to be strong indicators of the coagulopathic complications in patients [20]. These markers were reported to be more specific in comparison to other coagulation markers like D-dimers, fibrinogen, etc. [21]. Therefore, cumulatively, it can be inferred that a sensitive and efficient PoC PT sensing platform will be highly demanded by the healthcare sector in the coming years to cater to cardio-compromised and CAC-affected subjects. These coagulopathically challenged candidates, especially affected by cardiovascular aberration or COVID-associated coagulopathy, are prescribed anticoagulation or thromboprophylaxis therapy. The respective anti-coagulation therapy requires an efficient routine monitoring due to the narrow therapeutic window shared by anticoagulation drugs. A slight discrepancy in the anticoagulation drug dosage can lead to extreme life-threatening conditions resulting from thrombosis or hemorrhage. Thus, to avoid aberrations in anticoagulant dosage, the candidates under OAT or CAC critical care are recommended to perform frequent PT assaying at the respective point of care settings. A significant increase in usage of PoC home monitoring PT diagnostics both in Europe and US was noted with an increase in OAT or VKA prescription [22]. Thus, a continuous effort to devise an efficient PoC PT diagnostic is being made by the recent researchers. Several PoC platforms for PT diagnosis using optical, acoustic, electro-mechanical, or electrochemical methods through detection of changes in transmittance, viscosity, or electrical properties of whole blood or centrifuged plasma have been carried out. Examples of some of these commercially popular PoC PT/INR systems that have been awarded Food and Drug Administration (FDA) clearance include the CoaguChek System (by Roche Diagnostics Corp., Indianapolis, IN, USA), Harmony INR Monitoring System (by Lifescan, Inc., Milpitas, CA, USA), IN Ratio Prothrombin Time Monitoring System (by Hemosense, Inc., Milpitas, CA, USA), and ProTime Microcoagulation System (by International Technidyne Corp., Edison, NJ, USA), etc. [22]. The level of progressive research in the field of PoC PT platforms is supported through the demand driven market scenario of coagulation analyzers. A market valuing US$ 2481.7 million for coagulation analyzer has been reported in 2017 by Coherent Market Insights with a projected Compounded Annual Growth Rate (CAGR) of 9.7% over a forecast period of 2017–2025 [23]. Now these figures will definitely revise due to the pandemic and associated CAC cases. Around two decades ago in 1999, approximately three million Americans and about five million patients worldwide were administered anticoagulants regularly, where approximately 300 million PT-INR measurements were required in the U.S. alone [24]. The consequent global survey conducted for blood coagulation diagnostics reported over 200–800 million PT/INR tests conducted annually world-wide placing a huge burden on health care resources [24,25]. This shows the increase in demand of PoC PT/INR solutions and points out to a future need in coming years. The forecast statistics presented by Technavio (market research company) [26] prove that the prothrombin time (PT) analyzers hold maximum market share compared to other category of hemostatic analyzers, as shown in Figure 1. Thus, a sustained prevalence of a huge prospect in the sector of PoC PT/INR diagnostics can be inferred.

With an aim to provide an elaborate understanding of prothrombin time and related sensing platforms (PT assays), the following article enunciates the concepts of hemostasis and PT; clinical relevance of PT in thromboembolic complications and COVID-19 associated coagulopathy (CAC); lab-based PT/INR devices; point of care PT/INR assays; PT and associated protocols; and future prospects for PT sensing platforms and our conclusions about the future of this area.

## 2. Hemostasis and Prothrombin Time

**Figure 2:**
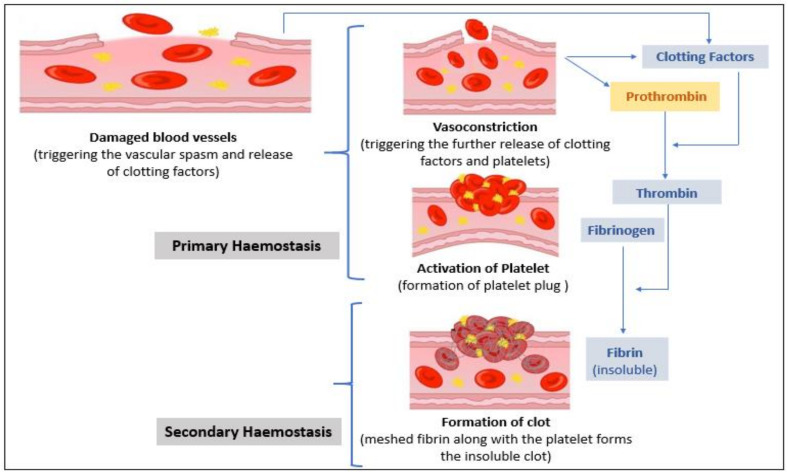
Schematic enunciating stages of hemostasis.

**Figure 3:**
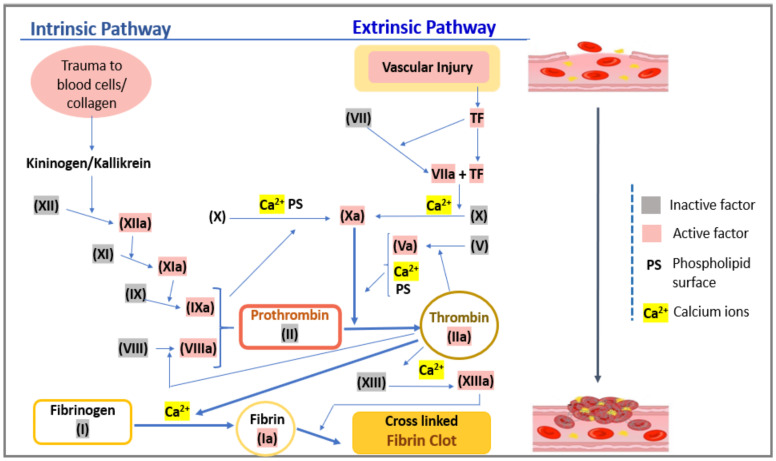
Schematic of Waterfall coagulation model.

Hemostasis is a crucial physiological process initiated by the body to constrict any kind of hemorrhagic condition encountered by the circulatory system (in case of internal or external vasculature injury). The process of hemostasis mainly involves the following phenomena: vasoconstriction, activation of platelets, and formation of blood clots, etc. [27], as shown in Figure 2. The process of vaso-constriction and activation of platelets is characterized by the contraction of blood vessels and the formation of a platelet plug, following which in the final step, the formation of the clot occurs. In this step, the thrombin cleaves fibrinogen into fibrin, which is further polymerized and crosslinked by a fibrin stabilizing factor (factor XIII) resulting in a stable mesh of fibrin. This mesh of fibrin further traps the chunk of platelet forming a stronger and long-lasting entity, stronger than the platelet plugs. Once the required amount of clot is formed to regulate the affected (trauma) region, cessation of the coagulation process and dissolving of excess fibrin is mandatory. Aberrations in regulatory mechanisms can lead to thrombotic tendencies. Thus, the check in the coagulation process is maintained by several regulators such as tissue factor pathway inhibitor (TFPI), protein C, antithrombin, and plasmin. Regulating factors antithrombin, protein C, and TFPI mainly act as an anticoagulant by degrading or limiting the action of several coagulating factors, thus further impeding the process of coagulation. Whereas, plasmin proteolytically cleaves fibrin into fibrin degradation products that inhibit excessive fibrin formation. Cumulatively, the entire process of reorganizing and regulating the process of clot formation is mainly performed by dissolving fibrin and is known as fibrinolysis [27,28]. In progression with the coagulation process, once the growing clot experiences any kind of contact with the endothelial tissue, the anticoagulation cascade is triggered to ensure the formation of unwanted thrombus generation. Before anticoagulation, the intricate process of coagulation is governed by several vitamins (e.g., Vitamin K) and several coagulating factors/proteins enlisted in Table 1 [27]. Coagulation factors are generally depicted using Roman numerals and its activated form with a suffix ‘a’. A series of factor-mediated protease (coagulation) reactions, as shown in Figure 3, involving cascaded production and activation of these coagulating factors (as enlisted in Table 1), leads to adequate production of fibrin, which seals and repairs the area of trauma. This set of related processes or phenomena is cumulatively referred to as the coagulation cascade/model. According to the Waterfall model of coagulation (as shown in Figure 3), the extrinsic pathway (path governing PT) of coagulation is triggered via damage to the blood vessel, leading to the diffusion of factor VII from the circulating stream. Factor VII, along with the tissue factor (TF) expressed on tissue-factor-bearing cells, leads to the formulation of an activated complex (TF-FVIIa), which further activates factor X (to Xa) [29]. Activated factor X (Xa) along with activated factor V (Va) form the prothrombinase complex, which essentially performs the conversion of prothrombin to thrombin. Finally, the thrombin formed acts on the fibrinogen to form fibrin by cutting it into fibrin filaments. Further with the help of the activated fibrin stabilizing factor (factor XIIIa), the fibrin filaments are polymerized into an insoluble clot along with platelets. This entire process of extrinsically mediated coagulation is clinically quantified as prothrombin time. Prothrombin time (PT) is a diagnostic assay that measures the length of time that the blood may take to clot through the set of reactions (as described above) involved in the extrinsic pathway. The normal range of prothrombin time is noted to be 11–13.5 s for healthy subjects. Thus, any aberration in the coagulation cascade (and factors such as II, V, VII, X, and Vitamin K [30]) is directly reflected in PT due to its direct correlation to the physiological clotting time. 

## 3. Clinical Relevance of PT in Thromboembolic Complications and COVID-19 Associated Coagulopathy CAC

As evident from the previous discussion, coagulation status (hemostasis) is a significant indicator of the physiological hemodynamics under trauma. Thus, prothrombin time and the associated INR serve as a potential biomarker for detecting several thromboembolic and hemorrhagic aberrations by correlating their deviations from the normal range. Several thromboembolic complications requiring sensitive PT assaying during diagnostic and therapeutic measures include atherosclerosis, atrial fibrillation (AF), myocardial infarction (MI), venous thromboembolism (VTE), and deep vein thrombosis (DVT) (often causing pulmonary embolism PE) [31,32]. The respective include both arterial and venous thromboembolism. Arterial thrombosis as in the case of AF (causing irregular beating of heart due to clots in the lining of the heart or valves), MI, and atherosclerosis (caused by clots in arteries or large arteries) often results in heart attacks [33]. In venous thrombo-embolic defects such as deep vein thrombosis, clots are formed in the veins deep within the body especially in lower legs [33]. These local thrombo-embolic complications often result in micro/macro circulatory embolism and lodging of clots in other parts of the physiological system such as the lungs, brain, kidney, etc., resulting in pulmonary embolism or stroke. Thus, the respective thromboembolic aberrations are mainly responsible for increasing incidence of cardiovascular disorders (CVD) such as atrial fibrillation, venous thrombosis, myocardial infarction, and related fatalities like pulmonary embolism, stroke, and transient ischemic attacks. These candidates with thromboembolic/cardio-vascular disorders are among the main candidates who are prescribed life-long oral anticoagulants (warfarin/coumarin dosages) to prevent intravascular clot formation [6,34]. During the oral anticoagulation therapeutic procedure, sensitive and periodic PT administration is essential, as the respective Vitamin K antagonists (VKA) are noted to share a narrow therapeutic window. It has been found through a survey that these anticoagulant drugs (warfarin) can have a variable effect on the hemostatic system, resulting in variations in the dosage of up to 120-fold between individual subjects [22]. Thus, the respective set of the population is strictly prescribed to perform frequent, periodic, and efficient PT assaying to monitor their coagulation status so that their further warfarin dosage levels can be decided. Failing to do this might lead to life-threatening situations like strokes (embolism), hemorrhagic issues, etc. In addition to the cardio-compromised sector (candidates under OAT), PT tests also include a sensitive population with pre-, peri-, and post-operative subjects [6] or subjects suffering from hemorrhagic conditions such SIC, hepatic/renal disorders, or being monitored for disease progressions such as dengue hemorrhagic fever [5] and the like. The respective hemorrhagic or critical coagulopathic conditions such as SIC and hepatic/renal disorders often result in multi-organ death syndrome (MODS), which may lead to fatality. Similarly, extreme cases of dengue hemorrhagic fever often lead to plasma leakage through gaps in the endothelium of the blood vessels, which can also lead to circulatory failure [5]. Thus, efficient PT diagnosis and optimal management of the respective therapy for hemorrhagic conditions like disseminated intravascular coagulation mediated SIC, congenital afibrinogenemia, Vitamin K deficiency, renal failure, and other bleeding disorders is a critical requirement. 

Apart from the several predefined well-known thromboembolic and coagulopathic aberrations discussed above, the growing prevalence of COVID-19 associated coagulopathy is evident with the increasing number of COVID-affected cases [7,12,35]. The June 2020 editorial by Lancet Haematology reported an incidence of about 49% of thrombotic complications in intensive care COVID-19 patients [12]. Although the main physiological manifestation of COVID-19 is an acute lung injury, along with the variety of symptomatic phenotypes like fever, dry cough, dyspnoea, and hypoxemia, there also exist several other life-threatening clinical manifestations at its critical stage. As the disease progresses towards a critical level, hemodynamic instability, acute respiratory distress syndrome (ARDS), rapid/multiple organ dysfunction syndrome (MODS), and coagulation dysfunction with major thromboembolic complications [18] are common. Severe Acute Respiratory Syndrome-Corona Virus (SARS-CoV-2) is reported to enter the cells by binding to the angiotensin-converting enzyme 2 receptors expressed on respiratory epithelial cells and other cells, inducing a florid host response characterized by dysregulation of inflammation and coagulation [36]. Since inflammation and coagulation are essential host defence mechanisms, aberrations in coagulation dynamics can be considered inevitable when the system is affected (with COVID). Dysfunctional coagulation dynamics are regarded as one of the primary causes of MODS and ARDS leading to systemic shutdown or death [7,37,38]. Thus, efficient monitoring of coagulation dynamics via coagulation biomarkers such as PT, D-dimers, fibrinogen level, and platelet count is almost mandatory for CAC therapeutic procedures. A survey corresponded by Long et al. at Tianyou Hospital of Wuhan University of Science and Technology between January and March 2020 found an increased D-dimer in 50 (43.5%) and fibrinogen in 74 (64.3%) patients on a total of 115 COVID confirmed cases [18]. Among these subjects, 23 resulted in deceased condition, from which 18 exhibited prolonged PT. Further, the receiver operating characteristic (ROC) analyses for mortality risk exhibited 0.937 as the area under the curve (AUC) for PT in critical cases, thus asserting PT as a strong independent predictor of the disease severity in addition to D-dimer in CAC cases [12,18]. Tang et al. also reported longer PT values in non-surviving patients with COVID-19 pneumonia in addition to higher D-dimer levels with fibrinogen degradation products (FDP) [8]. Additionally, a study presented by Trevisan et al. enunciated the significance of prolonged PT in predicting the level of hemorrhagic risk in critical COVID-affected cases [19]. A 93-year-old woman (already under OAT), in the post-acute phase of the disease, presented a marked elevation of PT-INR of 25.1, supporting a strong impact of the infection on the coagulative pattern and a state of critical coagulopathy [19]. This patient was provided an immediate venous infusion of three-factor prothrombin complex concentrate and vitamin K until PT-INR reduction started to happen. Thus, prophylactic measures along with efficient PT monitoring can be considered to be a mandate for critical COVID affected with CAC. Apart from PT itself, several other prothrombin markers such PT activity% (PT-act) and prothrombin fragment 1.2 were found to be strong and independent predictors of mortality risks in critical COVID-affected cases [21]. ROC derived for PT-act <75% by Luo et al. has provided an AUC of 0.905 compared to D-dimer with 0.816 and fibrinogen with 0.830, respectively [21]. Similarly, ROC analysis on 115 patients by Samkari et al. presented a superior specificity for prothrombin fragment 1.2 compared to D-Dimer for predicting the level of severity in COVID-affected thrombotic cases [20]. Clinically, in the initial infective stages of SARS-CoV2/COVID-19, it is often reported to induce a state of hypercoagulability, with both arterial and venous thromboembolism with a simultaneous progression towards coagulopathy [7,35]. The state of hypercoagulability often leads to microangiopathy, large vessel thrombosis, VTE, and DVT along with major thromboembolic complications, including pulmonary embolism in critically ill hospitalized patients [35]. Klok et al. studied 184 ICU patients with proven COVID-19 pneumonia (ICU patients of Dutch university hospitals on April 5, 2020), among which 139 (76%) were alive under thromboprophylaxis and 27% were diagnosed with VTE, 3.7% with arterial thrombotic events, and most of the cases with PE (81%) [13]. Hypercoagulability in the COVID affected often results in a state of micro/macro circulatory thromboembolism, creating a state mimicking DIC [38]. This results in a significant increase in D-Dimer and fibrinogen levels, as reported in certain initial critical cases. In a survey reported, 71.4% of non-survivors were found to meet the criteria for DIC stating DIC as a strong predictor for CAC [8]. The state of DIC often requires an immediate but precise thromboprophylaxis for the affected, or else there may be a life-threatening state due to thromboembolic complications or a state of extreme coagulopathy, followed by MODS. Following the state of hypercoagulability, leading to over-usage and exhaustion of coagulation factors, there is almost always a state of coagulopathy with low fibrinogen and platelet counts. The following hypo-coagulable state has also shown a state similar to SIC characterized with high PT (and low platelets and fibrinogen) and has been critical enough to cause MODS or death [12,35]. Usually, an SIC score >3 is considered a critical state of coagulopathy [38]. This state of coagulopathy requires a precise monitoring of PT status with continuous infusion of Vit K and prothrombin complex into the system. Consequently, although the prevalence of COVID-19 associated coagulopathy is evident and has been found to resemble SIC/DIC to a larger extent, its pathophysiology differs substantially from the other two and comprises a set of unique features of its own. VTE and arterial thrombosis are found to be more common in CAC compared to coagulopathy associated with SIC/DIC. Additionally, CAC is found to present similarities with hemophagocytic syndrome (HPS), antiphospholipid syndrome (APS), and thrombotic microangiopathy (TMA) [7]. Although CAC is reported to exhibit some similarities with several coagulopathic conditions, it has several unique characteristics compared to others, requiring further research in the understanding of the underlying pathophysiology of CAC [7]. The following state with undefined premises of CAC, exhibiting complex coagulopathic dynamics, creates a challenging state for its clinical diagnosis and prognosis. Thus, asserting that, efficient monitoring of coagulation biomarkers (such as PT, D-dimer, etc.) is an essential for effective prognosis and further understanding of the CAC-related dynamics. 

Cumulatively, it can be inferred that efficient monitoring of prothrombin time is a pre-requisite for performing efficient therapeutic procedures in an increasing set of the population, especially with cardiovascular disorders and the very recent (and critical) COVID associated coagulopathy state. Thus, the conventional and evolving methods of PT diagnosis are reviewed in the following sections. 

## 4. Lab-Based PT Monitoring

**Figure 4:**
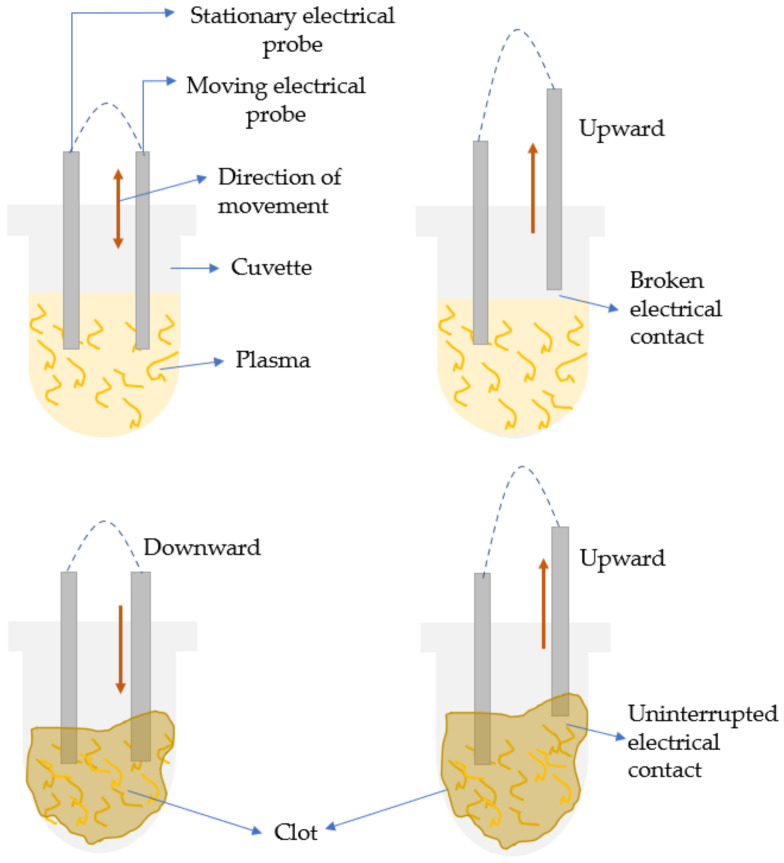
Schematic illustrating the conventional electromechanical form of coagulation detection method using moving electrical probe.

**Figure 5:**
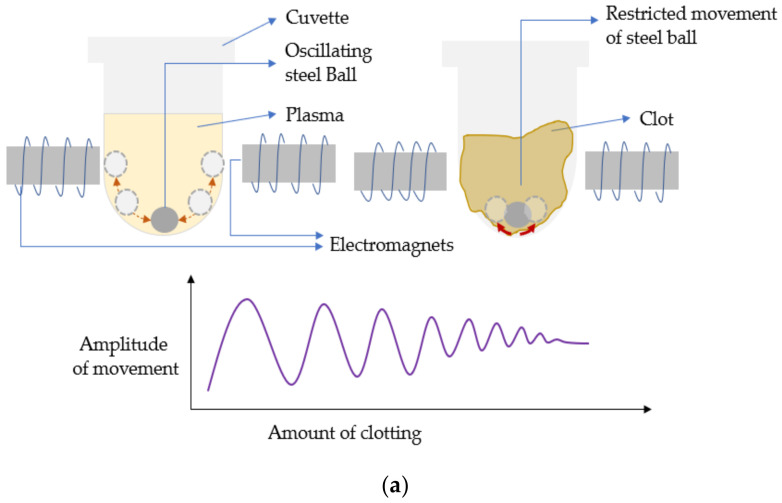

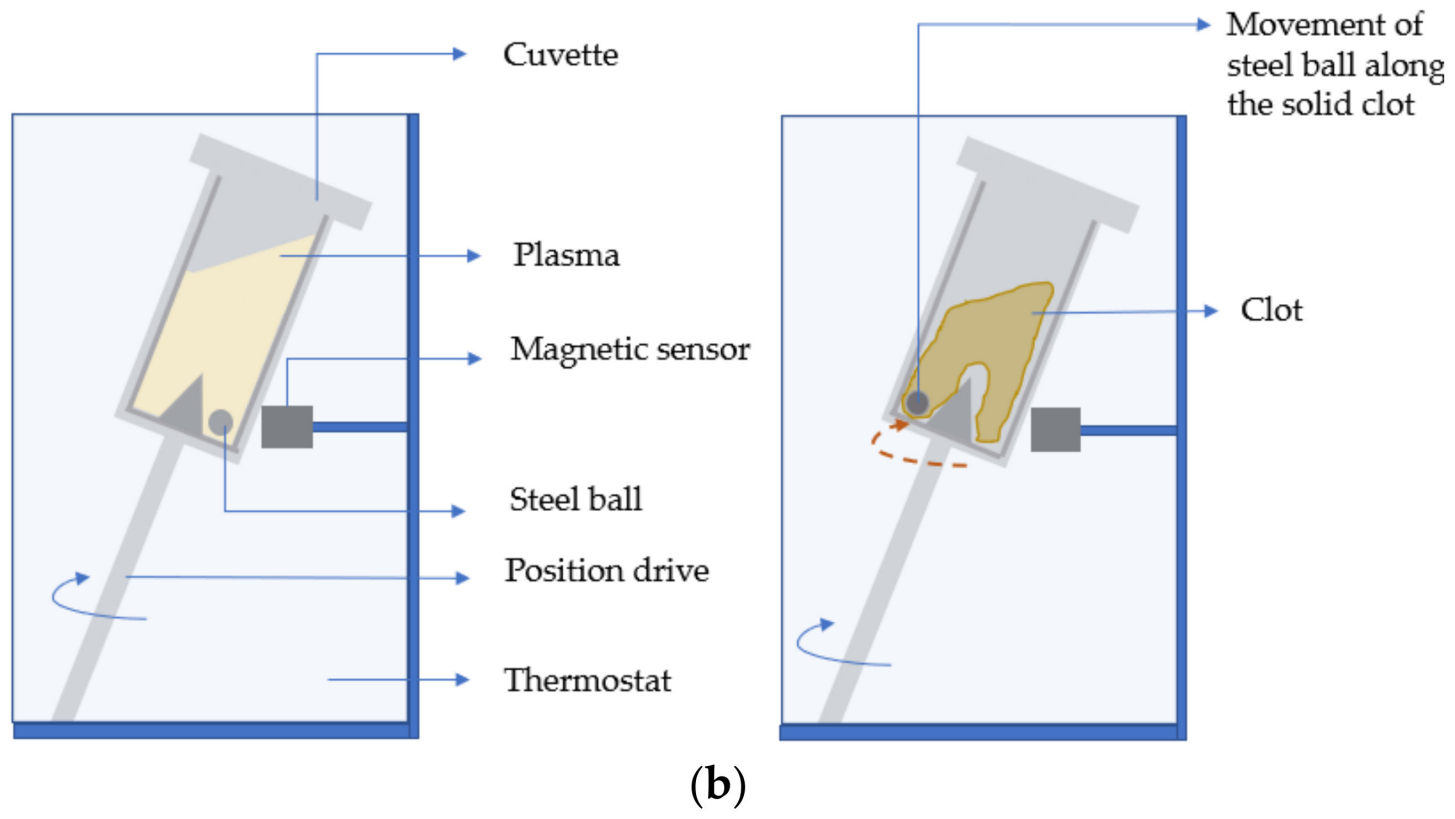
Schematics illustrating the conventional mechanical steel ball-based form of coagulation detection methods: (**a**) change in range of motion of magnetic steel ball and (**b**) break-in contact of steel ball with the magnetic sensors.

**Figure 6:**
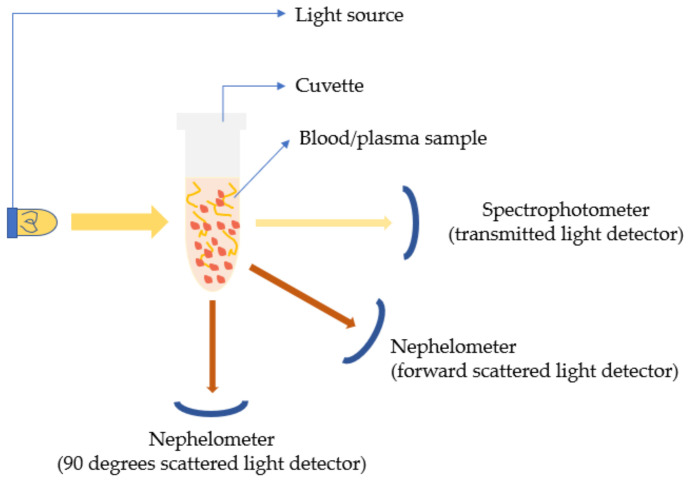
Schematic for conventional coagulation detection using optical methods (turbidimetry and nephelometry).

Most of the initial conventional hemostasis (PT) diagnostics were laboratory-based. The idea of the conventional coagulation time (CCT) detecting systems was based on an accelerated clotting phenomenon that was observed on real blood samples kept in glass containers containing some kind of biological tissues, e.g., placenta, etc. [39,40]. The very initial systems were based on visual observation and detection of fibrin and bulk changes in viscosity [40,41]. Eventually, the level of automation and technical sophistication grew, thus refuting the archaic visual detection methodologies and the associated inaccuracies. This increased level of automation resulted in many advantages such as increased flexibility, rapid/accurate results, cost reduction, etc. Conventionally, from the 1970s, the lab-based automated coagulation analyzers that existed were broadly categorized into mechanical and optical analyzers [30]. As detection methodologies were based on how the endpoint of clotting could be traced, they were categorized as endpoint detection systems or devices [42]. The devices employing mechanical methods as their detection principle were either electromechanical or magnetic. The mechanical methods were based on the detection of the increase in plasma viscosity, while fibrin formation was initiated. In electromechanical systems, the clot detection (such as BBL’s time-honored Fibrometer) [42] was traced using electrical probes that were put in contact with the electrolyte or the analyte of interest, i.e., the blood plasma. As the clot size grew, it resulted in the formation of a closed conducting electrical path, as shown in Figure 4. During the reaction, one of the electrical probes was made to move in and out of the solution at constant intervals. As the clot was formed, the fibrin plug formed an unbreakable continuous electrical contact between the fixed and the oscillating probes. The continuous electrical signal denoted the endpoint of the clot formation. In the magnetic steel ball-based clot detection method, monitoring the movement of a steel ball within the test solution with the help of a magnetic sensor was performed. The respective sensing could be implemented using two different methodologies [42]. Either the change in range of motion of the steel ball was recorded with the change of viscosity, or there was a recording of the break-in contact of the steel ball with the magnetic sensors as the steel ball gets dragged with the fibrin clot formed around it with induced cuvette rotation [42]. In one of them, an oscillatory motion is imparted to a magnetically coupled steel ball, immersed in the plasma-reagent solution, and consequently, its variation in motion is recorded. As the fibrin strands are formed, the viscosity increases, and eventually the oscillatory motions decrease to a predefined rate, notifying the clot formation as illustrated in Figure 5a. In the second variation, the steel ball is positioned in an inclined well whose position was detected by a magnetic sensor. In an unclogged state, as the well rotates, the ball continues to stay in its position on the incline. As soon as the coagulation increases, the ball is swept out of position, away from the sensor along with the solidified clot, causing a break in the circuit as shown in Figure 5b. Some popular CCTs like STA-R^®^ series (Diagnostica Stago Inc., Parsippany, NJ, USA) and Destiny PlusTM (Tcoag Ireland Limited, Wicklow, Ireland) implement the mechanical magnetic steel ball-based clot detection technique [42,43]. Similarly, there has been the existence of optical detection techniques. The conventional benchtop optical PT measuring devices use either photo-optical, nephelometric, or chromogenic principles to detect the changes in plasma viscosity [30]. Photo-optical instruments employ transmittance or turbidometry to detect coagulation [42]. Change in viscosity causes changes in the turbidity of the solution. Thus, if a light beam is made to pass through the respective solution, a difference in absorbed and transmitted light can be noted due to changes in the optical density of the solution. This method of sensing is called turbidimetry. The setup for these sensing platforms consists of a light source and a photodetector as shown in Figure 6. While recording the coagulation time, the level of transmittance continuously decreases with increasing viscosity. Opacity and viscosity of the sample increases due to gradual polymerization of fibrin. When the light of a specific wavelength is passed through the sample (plasma), the transmitted light is continuously recorded by the photodetector until a predetermined threshold level of minimum transmittance compared to the baseline is reached [42]. This is when the timer stops resulting in clotting time as the output. On the other hand, unlike turbidometry, nephelometry measures the intensity of the scattered light from the sample solution to detect the amount of clumping of particles. The process of clotting results in varying scattering of light in 180 or 90 degrees, which is then noted by a chronometer, as shown in Figure 6. The chronometer stops when the amount of scattered light reaches a specific predetermined intensity level, further noting the corresponding time as the coagulation endpoint. In nephelometry, the amount of scattered light increases with the increasing number of insoluble complexes. Nephelometric methods are contrast compared to turbidometric, as nephelometry notes the maximum level of scattered light as the endpoint of clot formation, whereas turbidometry measures the minimum level of transmittance compared to baseline as the endpoint. Nephelometry and turbidimetry are the main optical sensing phenomenon exercised for designing the conventional optical coagulation devices in comparison to other optical phenomena. STA-R^®^ series (Diagnostica Stago Inc., Parsippany, NJ, USA) [44], and Cascade^®^ series (Helena Laboratories, Beaumont, TX, USA) [45] are some of the popular devices with optical detection methodologies for observing the clotting phenomenon. In contrast to previously discussed optical detection methodologies, there exists chromogenic sensing, which employs optical sensing of the color emitted by the chromophores. Chromophores are color-producing substances usually used by chromogenic devices for quantifying the activity of the target element. Para-nitroaniline (pNA) is one of the common chromophores used by chromogenic assays. These chromogenic sensing platforms are comprised of a colourless synthetic oligopeptide substrate conjugated to the chromophores (pNA) [30]. The synthetic oligopeptide substrate is chosen such that it mimics the target sequence of the activated coagulation factor whose activity is to be determined. As the sample is introduced to the sensing platform and the coagulation is initiated, the coagulation protein cleaves the chromogenic substrate at the site binding the oligopeptide to the pNA. The process results in the release of free pNA. This action of releasing pNA produces a detectable yellow color proportional to the coagulation factor activity in terms of amount of pNA released. Further, the intensity of the yellow color emitted is measured using the photo detectors at 405 nm wavelength [42].

## 5. Point of Care PT/INR

The analyzing entity being blood, which is fluid and recognized to integrate well at the microscale, has caused an uncalled thrust for the use of microfluidic technology. The microfluidic devices not only exercise the dynamics of blood flow at the micro-level, but also provide results with a very low volume of analyte, resulting in rapid response. Although the flourishing micro-technology proved to be a boon, the main surge for the development of point-of-care PT/INR solutions has been caused due to the shortcomings faced by the conventional PT/INR. Apart from being bulky, expensive, and complex, its main shortcoming was high turnaround time. A high turnaround time for PT test could easily lead to life-threatening situations very often, as the candidates under PT test include a sensitive population: receiving anticoagulant therapy due to coagulation/cardiovascular disorders; under pre-, peri-, and post-operative evaluation; suffering from hemorrhagic conditions such as SIC or hepatic/renal disorders dengue hemorrhagic fever, or the COVID-19 affected experiencing post-infection CAC. Among the respective set of sensitive candidates, those under OAT (or with CVD) and the ones experiencing COVID-associated coagulopathy are the most critical ones requiring rapid and sensitive PT results during their diagnosis and thromboprophylaxis. The candidates under anticoagulation therapy (warfarin/coumarin dosage) share a narrow therapeutic window, thus requiring an in-hand rapid but sensitive PoC providing a quick PT diagnostic solution before its periodic anticoagulant dosage [6,34]. Inappropriate dosage may lead to a life-threatening situation like stroke (from embolism) or hemorrhagic issues [22]. Additionally, the large sector of the population affected by the recent pandemic coronavirus disease (COVID-19) is found to be experiencing immense thromboembolic and coagulopathic complications [7,35]. The pathophysiology of the COVID-associated coagulopathy is found to manifests a state of macro to microcirculatory embolism mediating towards coagulopathy (with prolonged PT), MODS, and associated fatalities [12,18,19,37]. Thus, there is a need for strict prophylactic measures along with rapid but sensitive coagulation assays such as PoC PT for efficient monitoring of coagulation processes.

In general, PoC devices developed using microtechnology consist of a disposable test card and an electronic test card reader unit. The test card generally consists of a sample area, microfluidic channels, and a testing zone functionalized with thromboplastin reagents to perform the PT tests. The underlying clot detection mechanism varies between devices. Existing PoC PT measurement devices can be broadly categorized by their sensing methods, i.e., optical, acoustic, or electromechanical, and electro-chemical. These sensing platforms detect the changes in transmittance, viscosity, or electrical properties of the whole blood or centrifuged plasma. This section mainly discusses some popular commercial PoC PT/INR products available along with the recent devices engineered by the researchers (yet to be made commercial) under the different detection schemes. It also elaborates on some attempts made by the scientific community to prepare devices that are easy and accessible to economically challenged sections of the population. An elaboration about the PoC PT measurement devices classified based on their sensing/detection methodologies is enunciated in the following subsections below.

### 5.1. Optical

**Figure 7:**
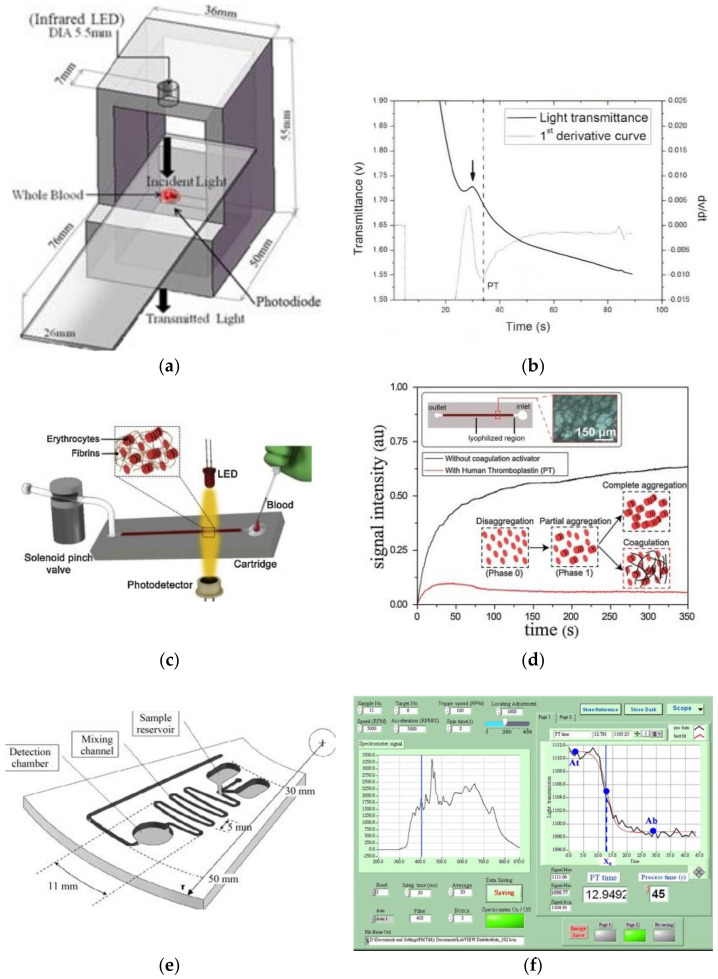
(**a**) Illustration of the transmission-based optical sensing module developed by Yang et al. (**b**) Plots for transmittance and its derivative for the whole blood during coagulation reaction. (Reprinted with permission from ref. [46]. Copyright 2013 Elsevier B.V.) (**c**) Schematic illustration of the coagulation time measuring platform developed by Isiksacan et al. in 2018. (**d**) Plots for the transmitted signal intensity of the whole blood w. r. t. time dispensed without any activator reagent and with PT reagent respectively. (Reprinted with permission from ref. [50]. Copyright 2018 Elsevier B.V.). (**e**) Illustration of the test cartridge of a microfluidic disc analyzer. (**f**) The graphical user interface for the PT test using a microfluidic disc analyzer (Reprinted with permission from ref. [31]. Copyright 2012 Elsevier B.V.).

In the optical category, there exist several commercial PoC PT/INR solutions that sense either the micro-optical characteristic changes or non-microscopic physical characteristics pertaining to the clotting behavior of the whole blood samples. Optical properties like transmittance, scattering, and refraction are generally categorized under micro-optical characteristics, and physical characteristics like flow behavior, distance, speed, acceleration, etc., are considered non-microscopic properties. The sensing mechanism of these instruments is based on the direct correlation of changes in the biophysical properties of blood (turbidimetry or viscosity) with the progressing coagulation process. Optical devices are one of the most preferred choices due to their relatively high versatility, low cost, and easy to engineer properties, and capability to provide highly sensitive and accurate measurements. The basic instrumentation associated with optical detection consists of a light source/emitter (LED), photodetector/photoresistor, and a sample placing platform or a test card with a reaction zone. Viscoelasticity and refractive index are the two main micro-optical properties used to develop optical fluidic assays. Changes in viscoelasticity are generally sensed via transmittance [46], absorbance, or scattering index (laser speckle rheology (LSR)) [25] of the analyte under coagulation. Secondly, the changes in refractive properties are sensed using the SPR (surface plasmon resonance) phenomenon. The transmittance-based devices follow Beer Lambert’s law, thus sensing the turbidimetric properties of the blood samples during the process of coagulation. The direct correlation of absorbance/transmittance with coagulation ensures efficient detection of the clotting time. Commercial PoCs such as Cascade POC (Helena Laboratories Point of care, USA) [45] and Coag-Sense™ PT/INR Monitoring System (CoaguSense Inc., Fremont, CA, USA) [47] are some popular POC coagulation analyzers (CA) built with transmittance based optical detection capabilities. Cascade POC (Helena Laboratories Point of Care, USA) comprises a test card with reconstituted paramagnetic iron oxide particles and other reagents into it. On placing the blood sample onto the reaction zone, the coagulation process is triggered. Further, the transmittance of light from the sample zone to the optical sensing unit is manipulated by varying the motion of the iron oxide particles under the influence of electromagnets. Once the analyte coagulates, cessation of the movement of iron particles happens, resulting in constant transmittance, further detecting the clotting time in seconds [45]. Coag-Sense™ PT/INR Monitoring System (CoaguSense Inc., Fremont, CA, USA) is another similar product devised to sense the transmittance during the coagulation process of the test sample. The test cartridge of this device consists of a sample area followed by a channel and a rotating spoked wheel, which draws the sample into a reaction well containing thromboplastin and performs mixing. A light beam is passed through the rotating spoked wheel containing the sample, and simultaneously the transmittance is monitored. Further, the formation of the clot causes an obstructed transmittance, which is detected by a photodetector present at the other end of the chamber to generate the equivalent PT/INR readings. Apart from the commercial devices, several transmittance-based designing attempts have been made by Yang et al. (2012, 2013) [48], Lin et al. (2014) [49], and Isiksacan et al. (2018) [50] in the recent past. Yang et al. (2012, 2013) proposed a portable coagulation device (Figure 7a) with an optical source, a receiving/detector module, a display module, a data transmission module, a timer, and a power supply integrated into a unit to perform data processing using the microsensor test card. During the process of coagulation, the transmitted optical signal is received by the receiving module and is further transduced into a voltage profile [48]. The point exhibiting a turn from minimum to maximum as shown in Figure 7b, by the first-order differential curve of the signal during coagulation is noted as the PT value. The mean relative error of the reported device in comparison to the standard manual PT method was reported to be less than 5% or 1 s. The same device with a few more design modifications was further tested for 167 whole blood samples, and it showed a success rate of 91.6%, which demonstrates the accuracy further [46]. Lin et al. in 2014 [49] also engineered an optical sensing module, but comprising a small-form-factor holographic optical element (HOE) mounted on a dual-stage seesaw actuator designed to sense the change in transmission efficiency and the total intensity of the reflected light. The following setup was utilized to measure PT by illuminating the sample using a 635 nm laser beam and further noting the transmittance and absorbance components from the reflector. The conversion of fibrinogen into non-solute fibrin was characterized by substantial changes in transmitted efficiency and total intensity of the reflected light. The respective sensing module provided an effective sensing platform using only 5 μL of whole blood/plasma. The coagulation time was determined within 6 min with a value of 4.12% CV (coefficient of variation). Whereas the gold standard analyzer Sysmex CA-1500, which uses transmittance speckle methodology, is reported to exhibit a turnaround of 31 min and CV of 5%. The detection of coagulation via measurement of changes in transmitted light was also utilized by Isiksacan et al. in 2018. However, the optofluidic point-of-care device by Isiksacan et al. implements the concept of sensing the erythrocyte aggregation as an indirect measure of investigating the PT, unlike the previous devices [50]. Illuminating the blood sample with near-infrared radiation and recording the changes in transmitted light intensity from the dispersed cell packs as they change to aggregated cell packs during the coagulation process is used as a basic measuring methodology in this device (as shown in Figure 7c,d). The efficacy of the device was validated by comparing the same with the conventional benchtop PT analyzer, which resulted in the goodness of the fit of 0.97 using linear regression. In addition to point (stationary) transmission-based PT analyzer, researchers have also investigated coagulation sensing using transmittance in dynamic platforms such as microfluidic disc analyzers. The tailoring of microchannel geometries as shown in Figure 7e also aided in efficient aliquoting, metering, plasma separation, decanting, and mixing of whole blood with various reagents [31,51,52], which aided to be a valuable addition to PoC PT sensing platforms. The evaluated PT/INR results of the respective platforms were correlated with that of the Sysmex CA1500, which resulted in a coefficient of determination of greater than 0.96.

**Figure 8:**
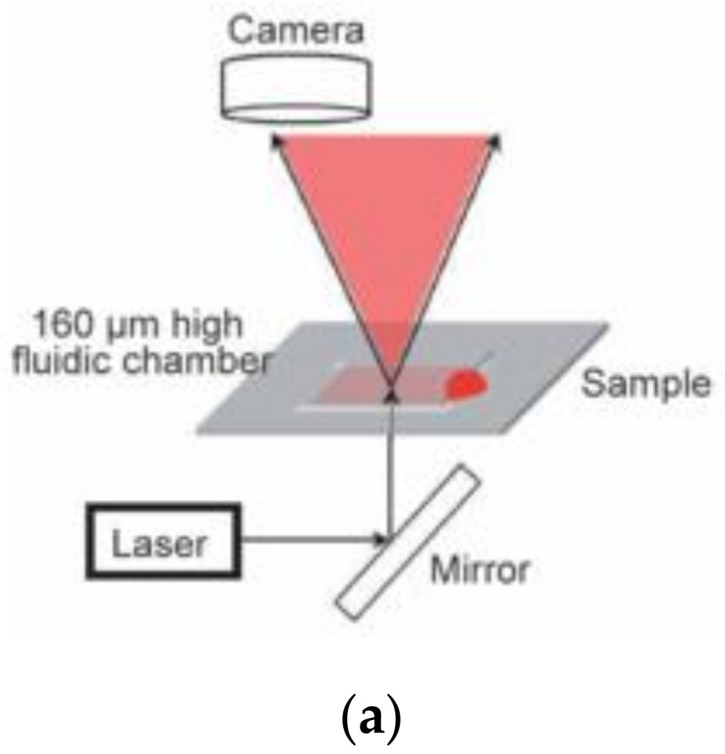

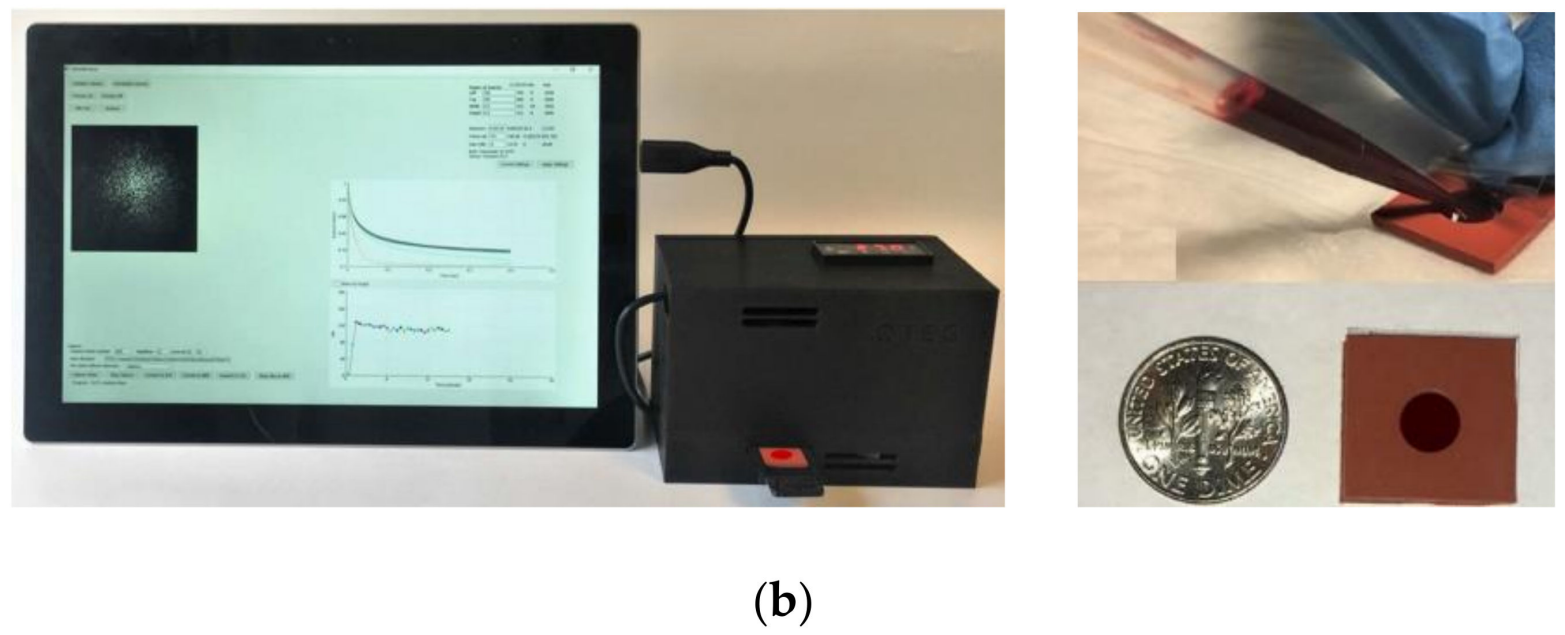
(**a**) Schematic of the optical setup, as engineered by Faivre et al. (Reprinted with permission from ref. [53]. Copyright 2011 SPIE Digital Library). (**b**) Photograph of the LSR based PT sensing module and test cartridge devised by Tripathi et al. (Reprinted with permission from ref. [25]).

Unlike transmittance, scattering is another important phenomenon in ray optics. In scattering, light rays get deflected from their initial straight path on striking an obstacle such as cellular components in blood. Change in cellular composition/configuration is inevitable due to aggregation and increase of fibrin mass during the process of coagulation. This causes changes in the scattering index of the analyte, which can be investigated to determine the coagulation time. Further, the required quantification of changes in the scattering index is predicted via laser speckle rheology (LSR). In LSR, the analyte consisting of a randomly distributed high number of diffusing objects (cellular components) is illuminated through a coherent light (laser) beam. As a result, each particle in the analyte diffuses the light, resulting in constructive or destructive interference phenomena between the diffused rays to form speckle images. The unbounded nature of the blood cells induces a residual/Brownian motion, resulting in a constantly changing speckle figure. This results in a “swarming” ensemble of speckle patterns with respect to time. The initiation of coagulation causes immobilization of cellular particles, resulting in a fixed speckle image that does not change anymore and is an indicator of the coagulation endpoint. Faivre et al. have devised a PoC optical setup as shown in Figure 8a [53] to measure the speckle patterns and their temporal fluctuations resulting from the laser-induced scattering of the coagulating analytes. Unlike the conventional method of measuring the temporal fluctuations in the resulting speckle pattern of scattered light, the device performs dynamic analysis of speckle patterns. The time of immobilizing of the cells (coagulation time) is duly determined by quantifying the differences between two consecutive images. Firstly, the correlation coefficient between the two consecutive images is calculated. Then, the derivative of the correlation coefficient w. r. t. time is derived. Further, the maximum of the derivative is accounted as the coagulation time, as the respective point denotes the maximum constitutional change in the sample under consideration. The results for coagulation time (PT/INR) derived by the respective sensing platform devised by Faivre et al. were found to be in very good agreement with standard laboratory methods. The calculated correlation results provided a determination coefficient of 0.9442. Similarly, Tripathi et al. (2017) [25] and Nadkarni et al. [54] (2019) also developed LSR based PT sensing platforms that are portable and battery-operated for rapid quantification of PT as illustrated in Figure 8b. The PT values are provided within seconds by measuring the temporal variations in viscoelastic modulus as the coagulation proceeds. In this setup, viscoelastic modulus (G) has been quantified using mean square displacement (MSD) of light scattering particles in a viscous medium. The PT is calculated as the time when a 2% drop of the maximum value of the first derivative of G(t) is obtained. The results of PT/INR using LSR have been found to be in close correlation and high concordance with standard PT values. In addition to these common micro-optical viscoelastic properties, i.e., transmission and LSR based system, Hayashi et al. have further investigated blood coagulation using SPR (surface plasmon resonance) phenomenon [55]. The sensing in this method is carried out by placing a 2 μL plasma at the inlet of a straight PDMS (poly-dimethyl-siloxane) microchannel and consequently monitoring the changes in flow velocity of the sample using SPR. The coagulation ability of different human plasma samples is quantified by observing the distinguishable flow velocity patterns caused by coagulation.

Apart from the methods described earlier and the microscopic optical method-based sensors, there also exist quite a few, non-microscopic flow behavior-based optical coagulation analyzers. MicroINR (iLine microsystems [56], ITC protime microcoagulation system [57], Hemochron Signature (ITC) [58], and GEM PCL PLUS Device are some of the early commercial devices that are based on optical detection of the sample flow behavior. The sensing platforms for these devices are built to sense the change in flow dynamics (distance, velocity, acceleration, etc.) of the coagulating media. Devices like Hemochron^®^ Signature, ITC protime micro-coagulation system, etc., detect the coagulation time optically by investigating the change in the fluid oscillation of a blood column as the coagulation cascade continues. Another commonly available analyzer, the MicroINR (iLine microsystems) [56], is available with an embedded machine vision algorithm that notes the change in position of the fluid in a microchannel. The positional data points are further transformed mathematically into speed and acceleration curves by the algorithm to determine the INR value. In flow-based analyzers generally, the sample blood dispensed into the microfluidic cartridge is initially made to homogeneously mix with the coagulating reagents with the help of multiple oscillatory pumps. Simultaneously when the mix is made to oscillate within the microcapillaries, the changes in fluid movement is tracked optically via optical sensors. The inherent coagulation process taking place within the blood sample results in gradual changes in the motion of the fluid providing a depiction for the coagulation process. Further, complete cessation in the movement ensures the end of coagulation. 

### 5.2. Acoustic/Electromechanical Resonators

**Figure 9:**
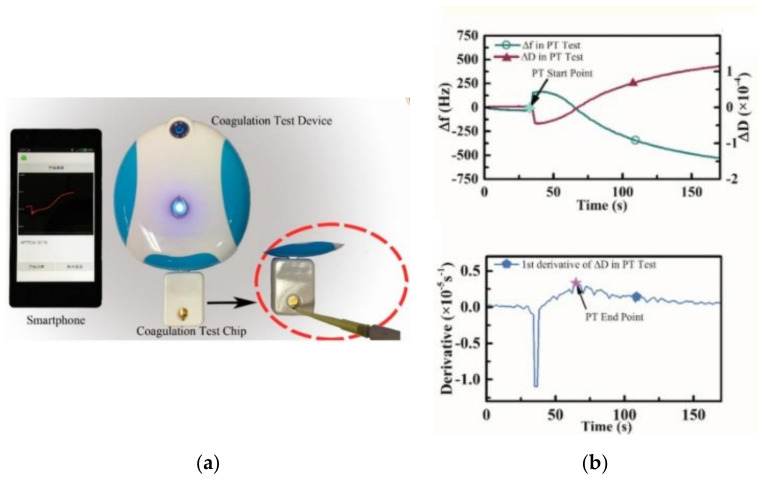

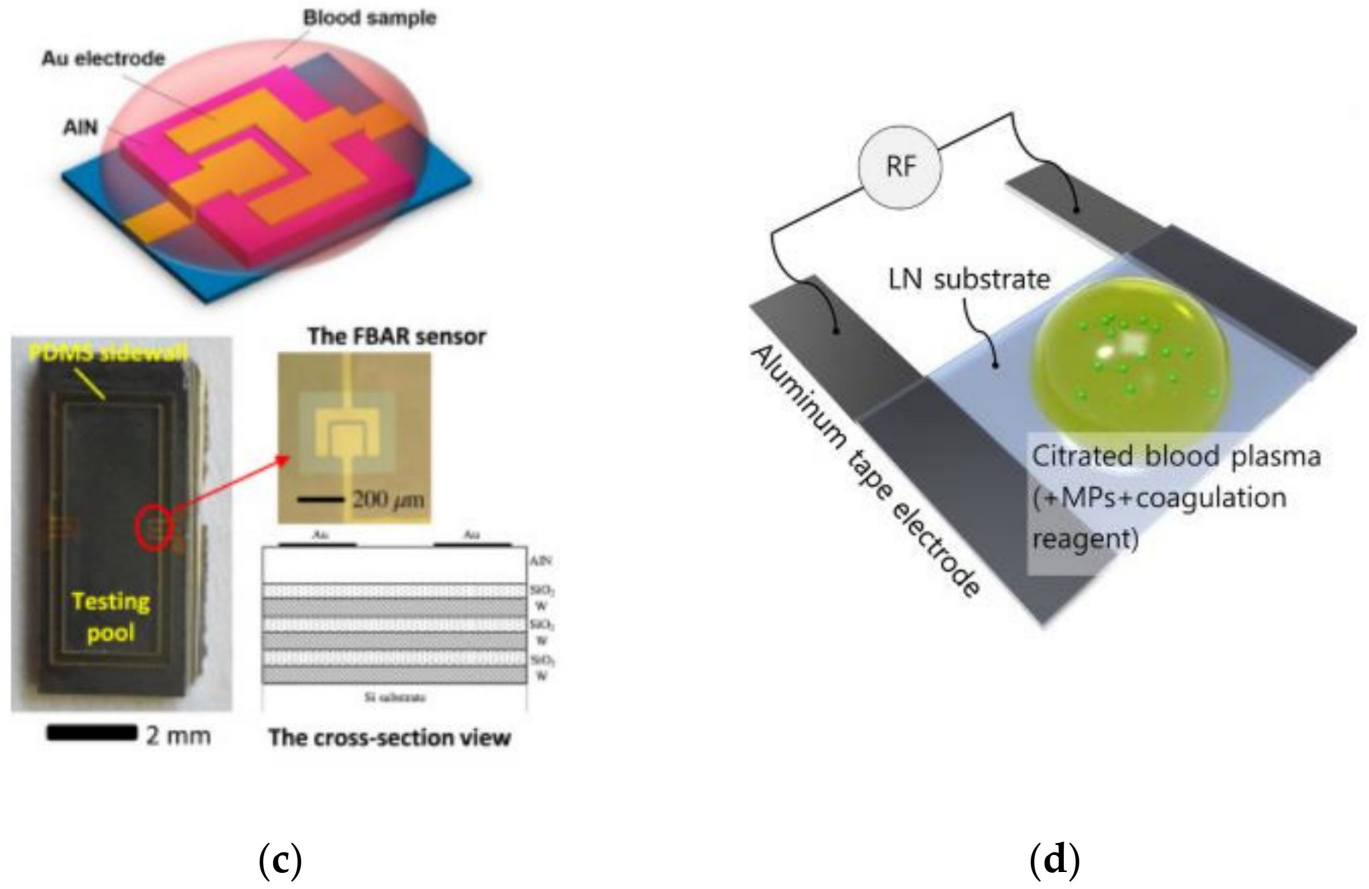
(**a**) Photograph of the smartphone/Bluetooth-based coagulation testing platform using quartz crystal microbalance dissipation method [61]. (**b**) Typical frequency and dissipation curves during PT measurements. (Reprinted with permission from ref. [61]). (**c**) Illustration and the microphotographs of the FBAR device. (Reprinted with permission from ref. [62]. Copyright 2017 Elsevier B.V.) (**d**) Illustration of the Lamb-wave based blood coagulation device embedded with piezoelectric substrate and aluminum tape electrodes. (Reprinted with permission from ref. [64]. Copyright 2018 Elsevier B.V.).

In acoustic or electromechanical sensors, blood coagulation is often sensed by analyzing mechanical parameters like change in blood viscosity by looking at the shift in resonance frequencies. Quartz crystal mass balance (QCM) resonator, surface acoustic wave (SAW) induced clotting, micro-electromechanical film bulk acoustic resonator (FBAR), and Lamb wave-based acoustics are some of the efficient acoustic measurement methodologies investigated for PT. In QCM acoustic sensors, frequency and dissipation are the two major properties that are sensed. Changes in mass loaded onto the sensor are depicted using a frequency shift, while the variation in viscoelastic behavior of the analyte attached to the layer oscillating with the QCM provides the dissipation factor. For driving the oscillations and reading them out of the quartz crystal, an oscillator circuit and a network analyzer are used in combination. Muller et al. (2010) [59], Hussain et al. (2015) [60], and Yao et al. (2018) [61] have investigated the successful and efficient detection of PT using QCM sensors and have nicely compared them to conventional coagulometers. Yao et al. extended the QCM sensing with a novel smartphone/Bluetooth-based testing platform for fast coagulation measurements as shown in Figure 9a,b. The device performed parylene-C coated quartz crystal microbalance (QCM) based dissipation measuring and analysis. The parylene-C coating provided a robust and adhesive surface for fibrin capturing, resulting in high R^2^ values for PT when compared to the standard CS-5100 hemostasis measuring system. Further, MEMS-based lateral field excited FBAR (Figure 9c) was introduced as a feasible tool for studying the rheology of blood by Chen et al. [62]. This resonator (FBAR) was excited by a lateral electric field and operated under the shear mode with a frequency of 1.9 GHz over a minuscule sample of 1 μL. The frequency response of the same was further studied to determine the changes in blood viscoelasticity and evaluate the coagulation time (prothrombin time) [62]. The measured PT values derived from the frequency differential presented provided a good linear fit (R^2^ = 0.99969) with the values of the commercial coagulometer. Unlike QCM and FBAR sensing methodologies, SAW and Lambda wave-based devices implement sensing of the velocity profile of whole blood rather than frequency measures of the coagulating media. These devices generally consist of fluorescent microspheres suspended into the analyte of interest. Once the coagulation is triggered, the fluorescent micro-spheres in the recalcified analyte experience changes in their translatory motion. Thus, the basic principle of these devices involves quantifying the coagulation time in terms of changes in the translational motion of fluorescent microspheres. Once the clot is fully formed, cessation in the movement of the microspheres occurs denoting the end point of clotting. SAW or the related acoustics were basically employed to instantly mix and re-calcify the citrated whole blood. Further, the quantification of clot formation kinetics was performed using image correlation analysis [63]. Similarly, Nam et al. [64] used Lamb wave to induce acoustic streaming of the fluorescent particles suspended in citrated plasma droplet via aluminum tape electrodes clamped onto the piezoelectric substrate (lithium niobate), as shown in Figure 9d. With progressing coagulation, the viscosity of the sample increases, resulting in a simultaneous decrease in acoustic streaming velocity. Further, the cessation of acoustic streaming of fluorescent particles suspended is noted to investigate the respective PT of the sample. PT results for the same have been further validated, and the comparative study with the commercial instruments provided a high determination coefficient of 0.9432.

### 5.3. Electrochemical

Blood, being a complex mixture of ionic species and insulating biomolecules, organically enables its interaction with electric potential and other chemical reagents to provide a detailed manifestation of its electrochemical dynamics. This level of intricate sensing at the molecular scale is believed to be efficiently realizable only when the sensing modalities have an electrochemical nature. Therefore, there exist several types of electrical and electrochemical PoC PT assays with high sensitivity of detection. Prothrombin time (PT) or the associated process of coagulation involves the change in surface charge, mobility, and biochemical state of the molecules. These physical factors further create changes in the bio-physical or electrochemical state of the analyte (blood) under consideration. Thus, the electrochemical assays are generally engineered to sense the changes in the electrical state of the analyte during the coagulation process either via (a) an indirect measure of the electrical activity caused due to cleaving of the peptide substrate pre-embedded on to the sensor surface, which is caused by some specific biomolecules or factors involved such as thrombin/fibrin, etc., or (b) by sensing the changes in the accumulative electrical state of the analyte in the form of impedance, conductivity, potential, or dielectric changes across the microelectrodes (in absence of any electroactive peptide substrates). CoaguCheck XS (Roche Diagnostics, Indianapolis, IA, USA) [65], Xprecia Stride^®^ Coagulation Analyzer (Siemens Healthcare GmbH, Norwood, MA, USA) [66], and i-STAT (Abbott Laboratories, Abbott Park, Illinois, USA) [67] are some of the popular commercially available amperometric electrochemical PT assays. These amperometric devices consist of lyophilized reagents constituting thromboplastin and a peptide substrate embedded onto the detection area. On the application of the sample, thromboplastin activates the process of coagulation, resulting in the formation of thrombin [65]. The enzyme thrombin further performs a cleaving action on the peptide substrate, generating an electro-active group. This further produces an electrochemical signal in the form of current at the electrodes embedded onto the test strips. This entire process of cleaving directly correlates to the amount of thrombin generated and the clotting time index, i.e., prothrombin time (PT). In addition to these commercially known devices, attempts were also made by some researchers such as Thuerlemann et al. (2009) [68] and Newman et al. (2010) [69] to engineer coagulation assaying based on substrate cleaving mechanism and amperometry.

**Figure 10:**
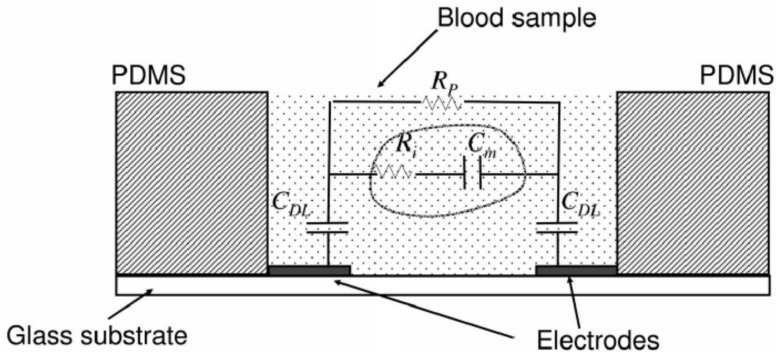
The equivalent electrical model of the whole blood, placed across two micro-electrodes embedded onto a PDMS micro-channel. (Reprinted with permission from ref. [72]. Copyright 2013 Lei et al.)

Along with these amperometry-based detection schemes, there also exists a substantial number of devices that sense the change of impedance, conductivity, or potential across the blood to detect the coagulation process. The sensing of the respective electrophysiological parameters of the blood is probably preferred, as the mechanism provides a direct and easily quantifiable measure of the electrophysiological changes in terms of resistance, impedance, or capacitance. The same is obtained by placing the blood analyte between a pair of microelectrodes, which results in an equivalent electric circuit with components like the plasma resistance (R_P_), cell interior resistance (R_i_), cell membrane capacitance (C_m_), and double-layer capacitance of electrodes (C_DL_) values, as shown in Figure 10 [70,71]. The equivalent model considered provides two parallel conductive pathways: one with impedance contributed from cells, and another contributed from the medium (plasma). Thus, the changes in the electrophysical state of the analyte during coagulation result in noticeable changes in resistive/impedimetric parameters of the plasma and cells (R_P_ and R_i_). The impedance change between the uncoagulated whole blood and the blood clot is significant, with R_P_ and R_i_ playing a vital role in intensifying the impedance over the clotting duration, whereas the C_m_ is known to dominate the impedance at the initial stages of coagulation. 

**Figure 11:**
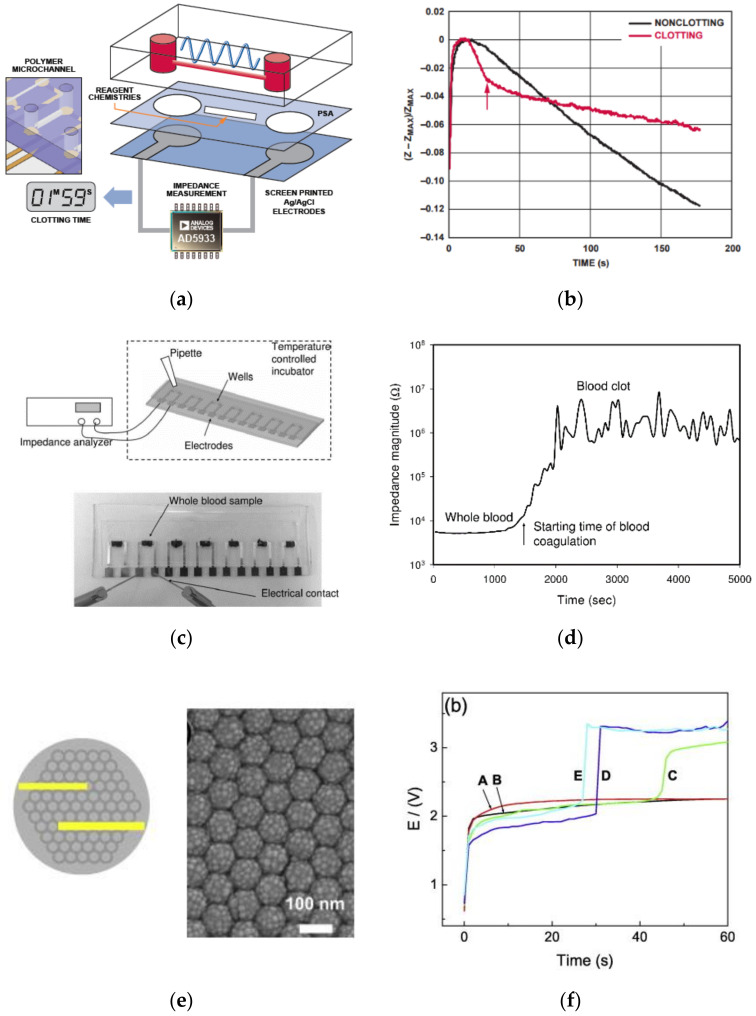
(**a**) Schematic of the impedance measurement system embedded with screen printed electrodes across the polymer microchannel designed to interface between the sample and the AD5933 instrumentation. (**b**) Impedance profiles for a nonclotting (black) and clotting (red) blood sample using AD5933 instrumentation (Reprinted with permission ref. [73]. Copyright 2008 Analog Devices, Inc.). (**c**) Illustration and the photograph of the real-time electrical impedimetric microfluidic chip developed by Lei et al. (**d**) Impedimetric profile for blood coagulation process at 1000 Hz. (Reprinted with permission from ref. [72]. Copyright 2013 Lei et al.) (**e**) Nanodots array along with the gold electrodes formed by sputtering, depicted using yellow lines and FE-SEM images of the same(surface). (**f**) Coagulation profiles in Au@nano-epoxy with A and B being blood sample and blood coagulation reagent, respectively, whereas C–E are the results of blood coagulation on Au@nano-epoxy along with coagulating reagents of 1, 3, and 5 μL, respectively. (Reprinted with permission from ref. [81]. Copyright 2009 Elsevier B.V.)

In impedimetric coagulation analyzers, the shift in the behavior of plasma and cell resistance during coagulation is quantified by noting the resultant impedance changes. During the process of coagulation, RBCs, platelets, and plasma are enmeshed by the fibrin fibers, forming aggregated cellular lumps or clots, leading to a drastic increase in the whole blood impedance/resistance compared to the normal whole blood impedance. In other words, changes in blood impedance are correlated to increased fibrin concentration in plasma. Some of the successful attempts to realize coagulation time using impedimetric signatures in microfluidic platforms have been made by Helen et al. (2008) [73], Farnam et al. [74], Lei et al. (2013) [72], and Chen et al. (2015) [75]. Helen et al. have developed a microfluidic test platform with screen-printed electrode accessorized with fully integrated single-chip impedance analyzer AD5933 (Figure 11a). In this design, an excitation voltage at constant frequency was provided, and recording of the differential impedimetric signals was carried out and correlated to the coagulation time, as shown in Figure 11b, to observe the coagulation. Lei et al. also developed a high throughput microfluidic chip (as shown in Figure 11c) with micro-fabricated gold electrodes using lithography for the investigation of the blood coagulation process (under temperature and hematocrit variations). A detailed theoretical background of the cellular dependence upon the coagulation process has been presented using the blood analyte electrical equivalent model, as enunciated previously in Figure 10 [72]. The frequency dependence of the excitation voltage on the output impedance (magnitude/phase) was also studied. Further, the impedance profiles at 0.1 V and 1000 Hz excitation voltage and frequency, respectively, have been obtained to quantify the related coagulation time parameter, as shown in Figure 11d. An electrochemical test strip developed by Chen et al. (assigned by Apex Biotechnology Corp., Hsinchu, Taiwan) was one of the novel electrochemical PT sensing platforms that existed in the market [75]. It comprised of hematocrit(HCT)% measuring zone along with PT measuring zones for hematocrit correction resulting in higher accuracy. In addition to hematocrit correction, the emphasis has also been provided to several other design parameters by other commercials to increase the sensitivity, such as through the controlled configuration of the printed electrodes [76], the geometry of the microchannels, the porosity of the substrate [77], etc. Zhang et al. (JOHNSON ELECTRIC S.A., Murten (CH)) have established that smooth deposition of electrode ink Parex PFO46 (thickness less than 10 μm) if carried out, could lead to the avoidance of disrupted fluid flow during the consequent impedimetric PT measurement [76]. Geometrical modifications, such as the concept of plurality of channels to avoid false results, were strongly emphasized by HemoSense INRatio [74,78,79] and INRatio/INRatio^®^2 (Alere Inc., San Diego, CA, USA) [76,80] PT test cards. Both the devices are classified as popular commercially available diagnostic devices that use impedimetric analysis to diagnose PT. They generally consist of a set of conducting electrodes or a noble metal electrode (Au/Ag/AgCl electrode set) on a disposable polymer strip, which can be used to monitor the electrochemical changes experienced by blood samples. Once exposed to the coagulating reagent with certain applied potential across the electrodes, the reaction progresses and leads to a continuous variation in electrical impedance till coagulation. A characteristic inflection point is identified by the system algorithm along the process, which is further denoted as the clotting endpoint [80]. The elapsed time, in seconds, from the initiation of the reaction until the endpoint is evaluated as the prothrombin time. The detection time and the coefficient of variation found in case of INRatio/INRatio^®^2 (Alere Inc., San Diego, CA, USA) were approximately ~1 min and 5.7%, respectively [80]. Although impedimetric measurements are quite comparable to the gold standard measurements for PT, Chen et al. (Apex Biotechnology Corp., Hsinchu, Taiwan) [77] have proved that reactance measurement may be more efficient compared to the typical impedance measurement method. The reactance measurement was made along with hematocrit correction by the proposed device for enhanced efficiency. Chen et al. also used porous substrates for improvement on the contact area of the analyte with the substrate. Consequently, it was inferred that the surface characteristic of the substrate is an important parameter in the regulation of the electrochemical measurements. Enhanced detection efficiency was also achieved by Lim et al. by surface manipulation of the substrate using nanodots [81]. The designed electrochemical assay for coagulation detection was designed using highly self-ordered nanodot sensors with a dot-height of 28 nm, as shown in Figure 11e [81]. It was observed that the process of blood coagulation is strongly influenced by surface roughness of an electrode. The basis for efficient detection is a result of a sudden increase in potential during the process of coagulation when a constant current is applied between the nanodot electrodes (as in Figure 11f). Voltage transient profiles have also been very recently used by Rudenko et al. to determine PT and hematocrit [82]. They have assayed several blood/coagulation parameters using an electrochemical microchip with two coplanar electrodes embedded onto the sensing zone that needs only <5 μL of blood. Further, the voltage transients exhibited by the sample during coagulation are measured and interpreted using a basic resistor-capacitor (RC) circuit and theory of fluid dynamics.

Unlike the resistive parameters like impedance, reactance, and voltage transients, there have been several attempts made by electrochemical assay designers where they have used current and conductivity to sense PT. HemoSense Inc. itself had its initial designs for coagulation assays based on conductivity profile, previous to impedimetric sensing assays [83]. Ramaswamy et al. (in 2014) and Lifei et al. (in 2018) have developed electrochemical microchips to sense conductivity across the microelectrodes, in order to measure the clotting time [84,85]. Very recently, dielectric spectroscopy was also explored for coagulation assaying by Maji et al. [86]. They have developed a clot chip with a sensor employing a three-dimensional (3D), parallel-plate, capacitive sensing structure embedded along with a floating electrode integrated into a microfluidic channel. Temporal variation of dielectric permittivity at 1MHz for human whole blood was accordingly noted, in which the peak corresponded to the onset of coagulation. 

### 5.4. Low-Cost Lateral Flow Assays for Performing PoC PT Diagnosis

**Figure 12:**
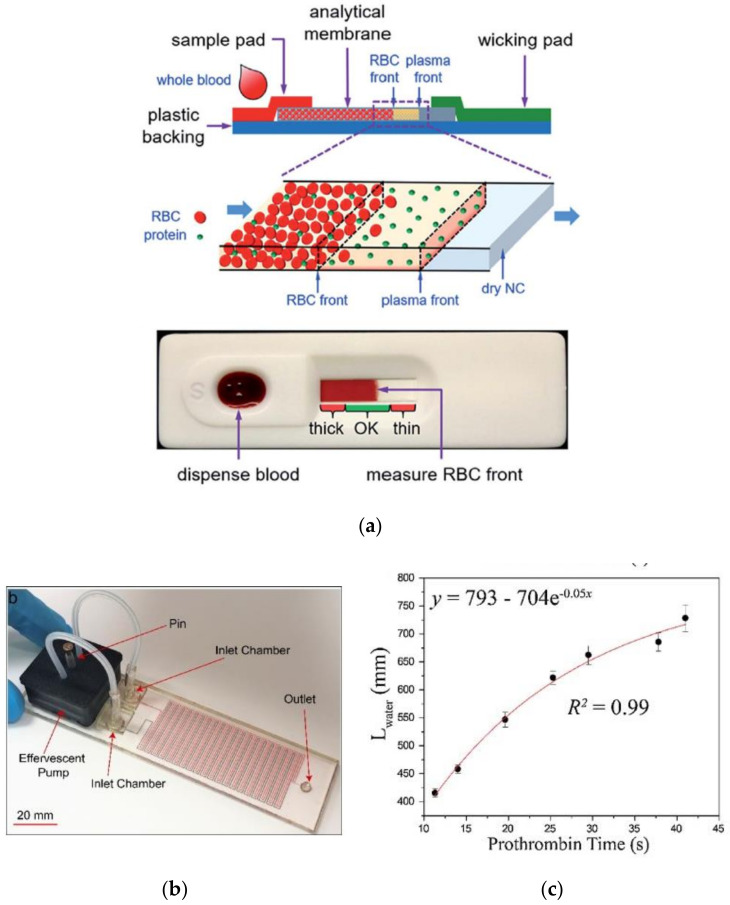
(**a**) Schematic illustration and photograph of the paper-based lateral flow assay (LFA) for coagulation time measurements engineered by Li et al. in 2018. (Reprinted with permission from ref. [92]. Copyright 2018 Royal Society of Chemistry.) (**b**) Photograph of the PT measurement device engineered by Guler et al. in 2018. (**c**) Correlation plot for conventional PT measurement versus water travel distance. (Reprinted with permission from ref. [32]. Copyright 2018 Elsevier B.V.).

Although there has been a significant advancement in Lab on Chip and MEMS-based sensing platforms, continuous research is persisted to optimize the cost and to simplify the operating methodology to facilitate home-based or point of care for the aged and rural population. These could be achieved via: (i) the usage of simple and low-cost fabrication methods, (ii) usage of low-cost substrate, (iii) usage of simplified detection methodology with a very adaptable and easy-to-use interface, etc. In order to achieve low-cost fabrication, paper-based devices are considered to be a viable and efficient option by microfluidic researchers. Unlike complex microfabrication methods like soft lithography, etc., simple fabrication methodologies like wax printing [87], inkjet printing [88], or screen-printing methods [89] are adopted in the fabrication of paper-based microsensors. In addition to the same, paper substrates can be considered as an exemplary option for LFAs for their biocompatible properties ensuring the interfacing of biochemical reagents. Additionally, the cost of active micro-pumps to ensure the desired microcapillary flow in MEMS sensors is also eliminated as in the case of paper devices as the medium itself supports the fluid flow via microcapillary action. Recently, a low-cost simple paper-based blood coagulation screening device as shown in Figure 12a has been developed by Li et al. using nitrocellulose membrane [90,91]. The device has nicely correlated the viscosity of the blood with respect to the distance travelled by the fluid (blood) providing a direct measure of its coagulability. In this device, when a drop of whole blood is introduced to the porous media (nitrocellulose membrane), an instantaneous capillary driven flow of the whole blood is executed, and the distance travelled by the RBCs through the membrane in a given time has been correlated to the blood clotting time. The authors have also reported a comparative of this process of detection with respect to the CoaData 2000 Fibrintimer, a clinical instrument available in the market, and have reported a nice performance correlation. In fact, it was found that the respective LFA proved to be slightly superior in detecting very weak or very strong coagulation as compared to CoaData 2000 Fibrintimer data. The correlation of hematocrit with that of coagulation time was, however, a factor affecting the PT results, which were further addressed and corrected by the author in the LFA device in their consequent studies [92]. The LFA device after hematocrit correction provided acceptable PT measures when compared and correlated to the results of the standard CoaguChek^®^XS coagulometer. The entire assay costs only 0.38 US dollars. Similarly, a low-cost LFA using PMMA (Poly-methyl methacrylate) cartridge has been developed by Guler et al. recently for prothrombin time measurement [32]. It is also a low-cost, easy-to-use LFA providing rapid PoC results. Thus, the respective LFA device can be considered to be promising for both lab- and home-based monitoring. The device comprises of: an integrated effervescent pump along with a two-channel test cartridge as shown in Figure 12b. A grid-based measurement scale is printed onto the cartridge to aid naked eye readout. The underlying principle of the device is to measure the distance traversed by the reference fluid(water) with known and constant dynamic viscosity until the sample fluids clots and stops flowing. The distance travelled by the reference liquid is further correlated to produce the PT for the sample liquid. The coefficient of determination between the PT values from conventional systems and the distance traversed by the reference fluid was found to be 99% using an exponential function as shown in Figure 12c. The respective exponential trend signifies that along with the increase in PT value, the distance travelled by the reference fluid also increases, but at a slower rate of time. Both the devices as proposed by Li et al. [93] and Guler et al. [32], as shown in Figure 12, provided an easy readout and cost efficiency as required for the targeted population. The respectives needed no additional electronic reader units. The fabrication of these sensing platforms was done using CO_2_ laser machining unlike complex lithography.

## 6. Prothrombin Time, Oral Anticoagulation Therapy, and Related Standardization Protocols for Designing/Calibrating and Usage of PT Assays (Especially at PoC Settings)

There exists a large but challenging prothrombin time assay sector under anticoagulation therapy with increased incidents of both cardiovascular disorder and COVID-19 associated coagulopathy. The research advances and increasing sophistication in designing PT diagnostics to achieve rapid but sensitive PT measurement are evident from the previous literature (as discussed in Section 4 and Section 5). Despite such advances, there exist aberrations and inefficiency in PT diagnosis. Inter-device variability due to variations in design methodology and coagulating reagents are some key challenges that lead to multiple sources of discrepancies at the designer level. Additionally, the lack of proper knowledge about the PT assaying protocol causes erroneous results at the user end. The key to achieving efficient PT diagnosis and management of the prevailing and upcoming challenges is to ensure strict follow-up and adherence to standardized protocols for PT assays on both the designer (calibration) and user level. 

As already discussed, prothrombin time (PT) is an assay that measures the clotting time of blood, mediated via an extrinsic pathway on the introduction of a clotting agent (tissue factor/thromboplastin). The measured value of PT may vary depending upon the amount and type of coagulating agent used to trigger the coagulation. The associated method of calibration used is also an important factor that might impact the prothrombin time results. Thus, from a global standardization point of view, the measured value (PT) is obtained by calculating the respective prothrombin ratio or an international normalized ratio (INR) as per Equation (1). International Normalized Ratio (INR) is the ratio of prothrombin time of the patient’s plasma sample to that of control plasma. The normalization is obtained considering the international sensitivity index (ISI) of the coagulating agent used. Quantitatively, the sensitivity of a chosen thromboplastin with respect to the reference thromboplastin (WHO standardized) is known as ISI. In general, ISI ranges between 0.94~1.4 for sensitive thromboplastins and 2.0~3.0 for less sensitive thromboplastins.

(1) $$\mathrm{INR}={\left(\frac{{\mathrm{PT}}_{\mathrm{test}}}{{\mathrm{PT}}_{\mathrm{normal}}}\right)}^{\mathrm{ISI}}$$

At the designer level, proper calibration of ISI and reduction in inter-device variability by gold standard calibration should be strictly followed. Conventionally, the ISI of table-top PT/INR systems were meant to be calibrated using the appropriate international reference preparation (IRP) method as standardized by World Health Organization (WHO) protocol. The procedure included fresh plasmas from 20 healthy subjects and 60 patients on oral anticoagulant therapy [22]. Consequently, a modified version of the WHO calibration protocol was introduced by Tripodi et al. for ISI calibration, which is the only recognized procedure for point-of-care capillary whole-blood PT assays [22]. In addition to calibration discrepancies, PT measurements are also impacted by the type of INR measurement principle used, resulting in inaccurate INR measurements. For example, Protime 3, was reported to provide lower INR values for samples with high plasma viscosity and high INR values for high hematocrit content [94]. The detection in Protime 3 is performed using the recording of back-and-forth movement of the capillary whole blood in the micro-channels tailored onto the test card. Further substantial coagulation resulting in obstruction of the channel and slowing of the blood flow, denotes the end of coagulation. Similarly, INRatio which works on impedimetric sensing rather than flow-based sensing also tends to provide higher INR values for samples with high hematocrit content [94]. In addition to the errors attributable to differences in test methodology, differences in INRs between PoC and lab-based PT diagnostics are reported. A clinical study as reported by Johnson et al. consisting of a PoC INR of 4.0 with a consequent clinical lab INR of 2.9, proves the presence of substantial discrepancies between clinical lab and PoC PT/INR results [95]. In general, PoC INR devices have been found to overestimate low INR values and underestimate high INR values [96]. Thus, comparison and calibration of test results for newly tailored PoC systems with the gold standard are recommended, implementing Bland–Altman plots and linear regression analysis protocols for an overall reduction in inter-device variability [95]. According to the study performed by Johnson et al., it was found that through the use of these algorithms, there has been a reduction of differences in the clinical lab and PoC assays by more than 43%. An approval for correlation standards of ≥95% for PoC INRs devices and correlation level of ±20% with clinical lab INR results was sanctioned by the Food and Drug Administration (FDA) [95]. Apart from the discrepancies attributable to ISI calibration and inter-device variability, there exist technical anomalies due to constitutional variations of blood resulting in inappropriate INR diagnosis. For example, deferred viscosity due to high/low hematocrit level (<25% or >55%), increased plasma viscosity, hyperbilrubinaemia (Bilirubin >170 μmol/L), hypertriglyceridemia (Triglycerides >5.6 μmol/L), hemolytic conditions, etc., are some of the aberrative scenarios leading to such discrepancies [94]. Similarly, the presence of heparin can also result in erroneous readings, as some INR PoC testing strips do not incorporate a heparin neutralizing reagent in them. Thus, a holistic approach for developing future micro-devices should be followed, which includes the intricate knowledge about both the physiological chemistry and dynamics of blood at molecular level. 

Simultaneously, at the user level, there existed minimal probability for handling errors due to the practice of assaying carried out by professionals at the clinic or hospital settings. But with increasing incidents of CVD/CAC, and the increase in candidates under OAT/thromboprophylaxis at home care settings, bring in additional challenges on the user interface design. PoC and home PT monitoring are reported to be widely practiced in Europe and the US. In Germany alone, over 100,000 patients performing patient self-testing (PST) in 2004 have been reported, and this level has increased several-fold since then [97]. A survey carried with 6417 participants by Heneghan et al. in 2012 reported a significant reduction in thromboembolic events in the self-monitoring group with a hazard ratio of 0.51 [98]. Although a significant decrease in cardiovascular emergencies is evident due to an increase in routine and rapid monitoring platforms, fatalities in candidates under OAT persist due to inappropriate dosage of anticoagulants resulting from lack of proper knowledge and training of PT/INR assay handling. Thus, at the user level, while engaging a patient in a self-management or self-testing system, candidates should undergo certain standardized guidelines issued by agencies like the Anticoagulation Self-management Association (ASA)/self-management program (SPOG), etc. [97]. Following this, a retrospective analysis presented by Bernardo, at the Cardiac Rehabilitation Centre in Bad Berleburg, Germany, reported 83.1% of patients trained with self-management programs were found to exhibit PT-INRs within desired therapeutic range [99]. Thus, a proper training and quality control measure/assessment for the self-monitoring sector of the population is highly needed and in fact should be mandatory to increase the awareness about proper handling during therapeutic measures to avoid measurement discrepancies. 

## 7. Discussion and Future Prospects

A wide variety of PT/INR assays implementing various transduction methodologies such as optical, mechanical/acoustics, and electrochemical means is continuously evolving. Research and implementation of various designing and standardization protocols are also being considered simultaneously. However, with an increased incidence of CVD and CAC-affected populations, rigorous research advances in PT assay design methodologies are inevitable. Although the previously designed PT assays have proven to be moderately efficient, a detailed and intricate comparative outlook among the devices and the design technologies is needed to be made to understand the technical advantages of one compared to the other. A summary of the recent advances and domain-specific commercialization spree are enlisted in Table 2 and Table 3 to provide some comparatives. Efficient development of a sensing platform, especially the PoC ones, requires an optimized choice of substrates, suitable micro-fabrication methodology, and design methodology complementing the desired efficiencies. Thus, for efficient and cost-effective PT design solutions, future modifications in the following domains are recommended: (a) suitable and efficient choice of transduction methodology, (b) suitable substrate choice for the sensing platforms (such as upcoming paper substrates), (c) optimization and usage of advanced microfabrication methodologies, etc.

As the discussion through the article elaborates various attempts made by the previous researchers to develop novel PT diagnostics to obtain an efficient sensing PoC platform, there exist numerous side-lined shortcomings for the same. The respective shortcomings must be brought out to enable bio-design processes ensuring a seamless detection methodology and protocol at chip-scale for studying coagulating behavior of blood samples. The interference of hematocrit percentage often proves to be an obstruction in measuring coagulation index, as seen more in optical assays conforming to colorimetric obstruction by RBCs, or in electrochemical assays in the form of cellular resistance, or perhaps in mechanical assays through a change of analyte viscosity due to the additions of cellular components, etc. As PoC mandates the usage of whole blood for rapid assaying, a well-calibrated hematocrit correction is recommended. As blood resembles a fluid with electrochemical properties, electrochemical PT sensors (impedimetric/capacitive/amperometric and dielectric) have proven to provide efficient results compared to their counterparts. Thus, in the near future, emphasis on developing electrochemical assays in conjunction with the use of the advancing molecular/nanoscience for developing such devices should be provided. Engineering devices using innovatively tailored nanoparticles/structures or nanotechnology for detecting detailed activities of the biomolecules/biofluids provide a large scope of exploration for future technologies within devices. As blood shares electronic properties at a micro level, more target- or factor-specific assays are mainly possible through the microelectronic route. With the emerging field of micro-electronics in flexible substrates (paper/plastic), an attempt to develop a sensitive but cheap sensor can be made possible. Most of the previously built sensors are developed in the PDMS/PMMA platform with gold/silver or platinum electrodes implementing complex fabrication and time-consuming methodologies such as lithography, screen printing, etc. With the advancing field of inkjet printing of conducting nanoparticles on flexible, biocompatible, and cost-effective substrates (such as paper), a huge reduction in cost can be made possible with no compromise in the sensitivity level of the engineered sensors. Therefore, the only unit adding expense to most devices pertains mostly to the electronic reader unit. There have been attempts made in the past by Guler et al., 2018 [32], Li et al., 2019 [90], and Hegener et al. [93] to develop completely cost-effective PT/INR solutions without the need for an additional electronic reader unit. The device developed by Guler et al. is a self-powered distance-based prothrombin time measuring device that can be read using naked eyes. The total cost of the device is approximated to be 1 USD. Similarly, Li et al. and Hegener et al.’s device was also a low-cost distance-based PT sensing platform developed using a paper substrate with a cost approximation of 0.38 USD per test card [93]. Thus, future researchers should attempt to overcome similar challenges to develop robust, sensitive, efficient, and cheap PoC PT/INR diagnostics capable of catering to both rural and urban sectors. 

## 8. Conclusions

The remarkable evolution of PoC PT diagnostics from conventional lab-based PT devices using several transduction methodologies (optical, acoustics/electromechanical, and electrochemical) across two decades has been studied and summarized. Considering the increasing prevalence of COVID Associated Coagulopathy in addition to the existing global burden of the population with vascular ailments and coagulation defects, development of PT diagnostics identifying with the ASSURED (affordable, sensitive, specific, user-friendly, rapid and robust, equipment-free, and deliverable to end-users) criteria is inferred to be essential. The increasing market share of the PoC PT diagnostics as projected by several coagulation market strategists has also been studied and is reported to surpass a CAGR of 9.7% by 2025. In addition to technical advances, physiological awareness related to coagulation dynamics has been implemented around the world but requires more rigorous spreading, especially in developing countries. The physiological dynamics of coagulation relating to various cardiovascular ailments are also gisted, and the impact made by coagulopathic and thromboembolic complications due to novel COVID-19 has also been enunciated consequently. The growing emphasis on thromboprophylaxis, OAT, and efficient PoC PT assaying platforms to achieve efficient prothrombin management is further described, and respective inferences are drawn for the importance of standardization protocols at both designer and user levels. Proper calibration requirements at the designer end subjected to types of thromboplastins and design methodologies are concluded to reduce inter-device variability. Proper training and quality control measures/assessment programs for the self-monitoring sector of the population are also mandated through the current research article. Further, advancements in the design methodologies, with the implementation of newer microfabrication methodologies, novel paper substrates, and evolving nanomaterials are recommended. Cumulatively, the requirement of optimization in design cost in addition to robust sensing methodology in future advancements of PoC PT diagnostics is emphasized through this article.

## Figures and Tables

**Table 1:** Several coagulation factors and their respective sources.

Coagulation Factor	Common Name	Source
Factor I	Fibrinogen	Liver
Factor II	Prothrombin	Liver
Factor III	Tissue factor and thromboplastin	Damaged tissue cells release tissue thromboplastin. Platelets release platelet thromboplastin.
Factor IV	Calcium ions	Bone and absorption through the lining of the small intestine
Factor V	Proaccelerin and labile factors	Liver and platelets
Factor VI	No longer used	N/A
Factor VII	Pro-convertin or stable factor	Liver
Factor VIII	Anti-hemophilic factor	Platelets and lining of blood vessels
Factor IX	Christmas factor	Liver
Factor X	Stuart prower factor	Liver
Factor XI	Plasma thromboplastin antecedent	Liver
Factor XII	Hageman factor	Liver
Factor XIII	Fibrin stabilizing factor	Liver

**Table 2:** Summary of the (PT/INR) measurement devices with their respective techniques and parameters.

Working Mechanism	Sensor or the Physical Phenomenon Sensed	Author/Developer (Year)	Volume and Blood Type	Sample Size (*n*)	Correlation Coefficient (r)/Coefficient of Determination (R^2^)	Comparative Gold Standard Device	Turnaround Time
Optical	Transmittance	Yang et al. (2013) [48]	~60 μL whole Blood (WB)	*n* = 26 relative error: 4.8 + 3.5% (>1 s)	r = 0.997,	Standard Coagulation Analyzer	<5 min
	Transmittance	Yang et al. (2013) [46]	~60 μL Both whole Blood (WB)	*n* = 167	whole blood INR r = 0.985, *p* < 0.001 plasmaINR(r = 0.948, *p* < 0.001)	conventional manual method and ACL TOP 700 bench-top coagulometer (Beckman Colter)	<5 min
	Transmittance	Isiksacan et al. (2018) [53]	50 μL WB	*n* = 21	R^2^ = 0.97	Conventional benchtop PT analyzer	<2 min
	Scattering	Faivre et al. (2011) [53]	10 μL whole blood		R^2^ = 0.94	Automated analyzerMDA II (Trinity Biotech, Bray, Ireland)	7 min
	LSR	Tripathi et al. (2017) [27]	40 μL whole blood	*n* = 60	R^2^ = 0.94 *p* < 0.001	laboratory PTLab/INRLab values	<30 s
	SPR	Hayashi et al. (2012) [55]	2 μL plasma				few mins
Acoustic	QCM	Muller et al. (2010) [59]	200 μL	2 samples *n* = 4	*p* < 0.05	Coagulometer	
	QCM	Munawar Hussain (2015) [60]	2.66 μL of plasma	*n* = 20		mechanical coagulometer (tCoag).	
	QCM	Yao et al. (2018) [61]	75 μL plasma		R^2^ = 0.961	CS-5100 hemostasis system	15 min
	FBAR	Chen et al. (2017) [62]	1 μL Both whole blood and plasma		R^2^ = 0.99969	commercial coagulometer	~12–15 min
	SAW	Santos et al. (2013) [63]	6 μL citrated whole blood				~3 min
	Lamb wave-based	Nam et al. (2018) [64]	PT reagent = 3 μL citrated blood plasma = 1.5 μL	*n* = 5	R^2^ = 0.9432	commercial instrument (STA-R Evolution)	
LFAs (optical)	Distance	Hegener et al. (2017) [93]	30 μL Capillary whole blood	*n* = 25/28	*p* < 0.05	CoaguChek^®^XS coagulometer	240 s
	Distance	Guler et al. (2018) [32]	50 μL whole blood	7 samples *n* = 3	R^2^ = 0.99	conventional benchtop (Sysmex, Siemens)	<2 min

**Table 3:** Some popular commercially available coagulation (PT/INR) monitoring devices.

Device Name/Manufacturer	Physical Phenomenon Sensed	Sample Type/Volume	Detection Time	Market Price
HemoSenseTM INRatio Prothrombin Time/INR	Impedance	Capillary whole blood 15 μL	<1 min	
Alere INRatio^®^2 PT/INR Monitoring System	Impedance	Capillary whole blood 9.5–15 μL	1 min	
CoaguChek XS Pro Meter (Roche Diagnostics)	Amperometry	Capillary whole blood 8 μL	1 min	$2014.00
Xprecia Stride^®^ Coagulation Analyser (Siemens Healthcare GmbH)	Amperometry	Capillary whole blood 6 µL		~$1428.00
i-STAT PT/INR by Abbott Labortories, USA	Amperometry	Capillary whole blood 20 μL	5 min	
ProTime InRhythm. (International Technidyne Corp, Edison, NJ, USA)	Pressure driven clot detection technology	Capillary whole blood ~13 μL	<1 min	
Coumatrak (Biotrack, USA)				
Cascade POC (Helena Laboratories Point of care, USA)	Photodetection (transmission)	30 to 35 µL	<1 min	
Coag-Sense™ PT/INR Monitoring System (CoaguSense Inc., Fremont, CA, USA)	Optical (transmission)	minimum 10 µL		$56.95–950.00
MicroINR (iLine microsystems	Optical (blood flow behavior)	3 μL		$ 442.79
Hemochron Signature (ITC)	Optical detection (oscillatory flow behavior based)		Few minutes	$495.00

## Data Availability

All relevant figures and data borrowed from various publishers and sources have been duly permitted by the concerned and is available as “reprinted by kind permission from the referenced authors of the respective articles”.
